# YTHDF2 correlates with tumor immune infiltrates in lower-grade glioma

**DOI:** 10.18632/aging.103812

**Published:** 2020-09-27

**Authors:** Xiangan Lin, Zhichao Wang, Guangda Yang, Guohua Wen, Hailiang Zhang

**Affiliations:** 1 Department of Cancer Chemotherapy, Sun Yat-Sen Memorial Hospital of Sun Yat-Sen University, Guangzhou 510000, China; 2 Department of Cancer Chemotherapy, Zengcheng District People’s Hospital of Guangzhou, Guangzhou 511300, China

**Keywords:** YTHDF2, lower-grade glioma, biomarker, tumor-infiltrating, prognosis

## Abstract

Immunotherapy is an effective treatment for many cancer types. However, YTHDF2 effects on the prognosis of different tumors and correlation with tumor immune infiltration are unclear. Here, we analyzed The Cancer Genome Atlas and Gene Expression Omnibus data obtained through various web-based platforms. The analyses showed that YTHDF2 expression and associated prognoses may depend on cancer type. High YTHDF2 expression was associated with poor overall survival in lower-grade glioma (LGG). In addition, YTHDF2 expression positively correlated with expression of several immune cell markers, including PD-1, TIM-3, and CTLA-4, as well as tumor-associated macrophage gene markers, and isocitrate dehydrogenase 1 in LGG. These findings suggest that YTHDF2 is a potential prognostic biomarker that correlates with LGG tumor-infiltrating immune cells.

## INTRODUCTION

Despite therapeutic advances in recent years, cancer still ranks as a leading cause of death [1]. The Cancer Genome Atlas (TCGA) program and Gene Expression Omnibus (GEO) data, which provide important information for further understanding of tumor biology, are available to users via multiple web-based platforms ([2–11]). This knowledge is essential and has already been incorporated into clinical practice, improving our ability to diagnose, treat, and prevent cancer.

Immunotherapy based on cytotoxic T lymphocyte-associated antigen 4 (CTLA4), programmed death-1 (PD-1), and programmed death ligand-1 (PD-L1) inhibitors has emerged as an effective treatment in melanoma and non–small-cell lung carcinoma [12, 13]. As noted in several studies, tumor-infiltrating lymphocytes, such as tumor-associated macrophages (TAMs), play an important role in patient prognosis and the efficacy of immunotherapy [14–17]. Some markers have been identified as effectors of immunotherapy [18–20]. However, current immunotherapy strategies have shown poor clinical efficacy in other cancers [21–23]. Therefore, identifying efficacious immune-related therapeutic targets in cancers is urgently needed.

m6A is a prevalent internal mRNA modification [24, 25] and plays an important role in cancer progression [26] and immunoregulation [27]. m6A modification is regulated by “writers” (m6A methyltransferases, such as methyltransferase-like 3 [METTL3] and methyltransferase-like 14), “erasers” (m6A demethyltransferases, such as fat mass and obesity-associated [FTO] and alkB homologue 5, RNA demethylase), and “readers” (effectors recognizing m6A, such as three YTH domain proteins [YTHDF1–3]) [28]. m6A modification (deletion of METTL3 or YTHDF2) controls the innate immune response to infection by targeting type I interferons [29]. m6A modification by FTO increases melanoma growth and decreases response to anti–PD-1 blockade immunotherapy [30]. METTL3-mediated mRNA m6A methylation promotes dendritic cell (DC) activation and function [31]. YTHDF1 shows anti-tumor immunity in DCs [32]. YTHDF2 sequesters m6A-circRNA and is essential for suppression of innate immunity [33]. In addition, YTHDF2 plays cell type-specific roles in lytic viral gene expression during Kaposi's sarcoma-associated herpesvirus infection [34]. YTHDF2 is a functional m6A-specific reader protein that mainly regulates stability of mRNA [35]. A previous study showed that YTHDF2 expression was regulated by miR-145 in hepatocellular carcinoma (HCC) cells [36]. Moreover, YTHDF2 may function as a tumor suppressor to inhibit cell proliferation and growth in HCC [37]. In addition, YTHDF2 acted as a tumor oncogene to promote prostate cancer cell proliferation and migration [38]. Interestingly, it has been found that YTHDF2 plays dual roles in pancreatic cancer cells by promoting proliferation and inhibiting migration and invasion [39]. Therefore, the roles of YTHDF2 in cancer remain elusive, especially regarding tumor-immune interactions.

In this study, we analyzed YTHDF2 expression and its correlation with the prognosis of cancer patients via a pan-cancer analysis using various web-based platforms. We also investigated the relationship between YTHDF2 expression and tumor-infiltrating immune cells (TIICs) in various cancers. Moreover, we analyzed the correlation of YTHDF2 with isocitrate dehydrogenase 1 (IDH1) in LGG. Finally, we performed the enrichment analysis of YTHDF2 in LGG. These results shed light on the important role of YTHDF2 in LGG and provide an underlying mechanism between YTHDF2 and tumor-immune interactions.

## RESULTS

### YTHDF2 expression in cancer

**Figure 1:**
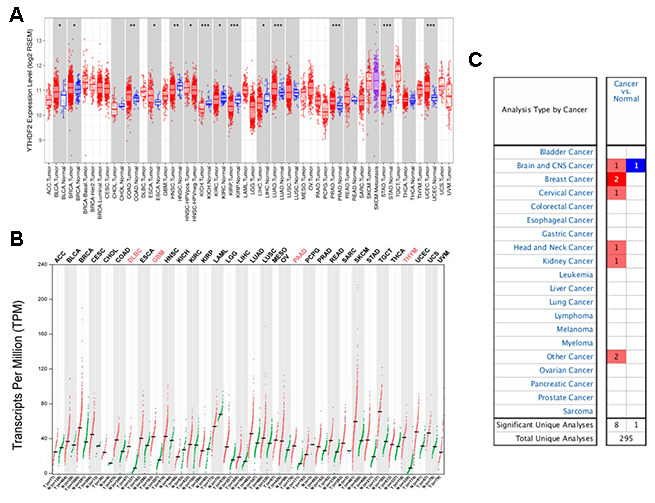
****YTHDF2 expression in different types of human cancers were determined with (**A**) the TIMER, (**B**) GEPIA, and (**C**) ONCOMINE databases. ***P<0.001, **P<0.01, *P<0.05.

We used the Tumor Immune Estimation Resource (TIMER) database to study differences in YTHDF2 expression in tumor tissues and adjacent normal tissues. Figure 1A shows that YTHDF2 expression was substantially higher in BLCA (bladder urothelial carcinoma), breast invasive carcinoma, colon adenocarcinoma, esophageal carcinoma, LUAD (lung adenocarcinoma), stomach adenocarcinoma, prostate adenocarcinoma, and UCEC (uterine corpus endometrial carcinoma) tissues than in adjacent normal tissues. However, YTHDF2 expression was lower in head and neck squamous cell carcinoma, KICH (kidney chromophobe), KIRC (kidney renal clear cell carcinoma), kidney renal papillary cell carcinoma, and LIHC (liver hepatocellular carcinoma) tissues than in adjacent normal tissues. YTHDF2 expression was not expressed substantially between cholangiocarcinoma, lung squamous cell carcinoma, READ (rectum adenocarcinoma), and thyroid carcinoma tissues and adjacent normal tissues. Unfortunately, no data were available on YTHDF2 expression in adjacent normal tissues for the following cancers: adrenocortical carcinoma, DLBC (lymphoid neoplasm diffuse large B-cell lymphoma), GBM (glioblastoma multiforme), LAML (acute myeloid leukemia), LGG (lower-grade glioma), mesothelioma, OV (ovarian serous cystadenocarcinoma), PAAD (pancreatic adenocarcinoma), pheochromocytoma and paraganglioma, SARC (sarcoma), skin cutaneous melanoma, testicular germ cell tumor, thymoma, uterine carcinosarcoma, and uveal melanoma.

To provide a more comprehensive evaluation of YTHDF2 expression in cancers, we used the online database Gene Expression Profiling Interactive Analysis (GEPIA) to compare YTHDF2 expression across 33 TCGA cancer types and in TCGA and GTEx normal tissues. Figure 1B shows that YTHDF2 expression was elevated in many cancers, especially DLBC, GBM, PAAD, and THYM.

We then used the ONCOMINE database to compare YTHDF2 expression in human cancer and corresponding normal samples (Figure 1C and Supplementary Table 1). Supplementary Table 1 ([40–46]) shows YTHDF2 datasets in human cancers. YTHDF2 expression upregulated in anaplastic oligoastrocytoma, with a fold change of 2.433, and downregulated in glioblastoma, with a fold change of –2.762. In addition, YTHDF2 expression upregulated in the other cancers, with a fold change from 2.038 to 11.69.

**Table 1:** Univariate and multivariate analysis of association of YTHDF2 and prognostic factors with overall survival in LGG, LIHC and SARC.

**Parameter**	**LGG**				**LIHC**				**SARC**			
	**Univariate analysis**		**Multivariate analysis**		**Univariate analysis**		**Multivariate analysis**		**Univariate analysis**		**Multivariate analysis**	
	HR (95% CI)	P-value	HR (95% CI)	P-value	HR (95% CI)	P-value	HR (95% CI)	P-value	HR (95% CI)	P-value	HR (95% CI)	P-value
Age	1.058 (1.043-1.073)	***	1.057 (1.041-1.073)	***	1.01 (0.997-1.024)	0.139			1.019 (1.003- 1.034)	*	1.019 (1.004-1.033)	*
gender (male)	1.092 (0.765-1.557)	0.629			0.816 (0.573-1.163)	0.26			0.87 (0.584 -1.297)	0.494		
raceBlack	4939923 (0-Inf)	0.993			1.542 (0.656-3.622)	0.321			1.073 (0.130-8.821)	0.948		
raceWhite	3286235 (0-Inf)	0.993			1.300 (0.893-1.894)	0.172			0.788 (0.108-5.750)	0.814		
Tumor Purity	0.562 (0.25-1.261)	0.162			2.07 (0.901-4.759)	0.087			2.003 (0.723-5.551)	0.181		
B cell	830.428 (54.364-12685)	***	3.450 (0.011-1042.915)	0.671	0.864 (0.053-13.978)	0.918			0.224 (0.006-8.9)	0.426		
CD8+Tcell	19943.51 (1320.611-301181.6)	***	5.782 (0.005-6512.228)	0.625	0.515 (0.053-5.035)	0.569			0.677 (0.039-11.68)	0.788		
CD4+Tcell	47.835 (6.336-361.158)	***	0.062 (0.000-188.625)	0.497	11.602 (0.483-278.815)	0.131			0.016 (0.001-0.436)	*	0.016 (0.001-0.425)	*
Macrophage	296.664 (52.011-1692.124)	***	851.361 (15.430-46973.874)	**	22.634 (1.631-314.017)	*	23.940(0.535-1070.315)	0.101	0.41 (0.05- 3.368)	0.407		
Neutrophil	881.918 (66.197-11749.39)	***	0.016 (0.000-49.469)	0.314	486.294 (2.269-104217.1)	*	0.299(0.000- 2654.334)	0.795	0.003 (0- 3.517)	0.107		
Dendritic	10.994 (4.24-28.506)	***	3.874 (0.095-157.366)	0.474	1.74 (0.54-5.612)	0.354			0.359 (0.088-1.475)	0.156		
YTHDF2	2.749 (1.697-4.453)	***	1.984 (1.104-3.565)	*	2.194 (1.334 -3.608)	**	2.094(1.270- 3.454)	**	3.024 (1.725-5.302)	***	3.013 (1.720-5.277)	***

LGG, Brain Lower Grade Glioma; LIHC, Liver hepatocellular carcinoma; SARC, Sarcoma; YTHDF2, YTH N6-methyladenosine RNA binding protein 2; Cor, R value of Spearman’s correlation; None, correlation without adjustment. Purity, correlation adjusted by purity.P-value Significant Codes: 0 ≤ *** < 0.001 ≤ ** < 0.01 ≤ * < 0.05.

### Prognostic value of YTHDF2 in cancer

We investigated the impact of YTHDF2 expression on survival rates by using the PrognoScan database. The relationships between YTHDF2 expression and prognosis in different cancers are shown in Supplementary Table 2. YTHDF2 expression substantially impacted the prognosis of four cancer types, including brain, breast, colorectal, and soft tissue. However, the impact of YTHDF2 on survival was conflicting in two independent breast cancer cohorts.

**Figure 2:**
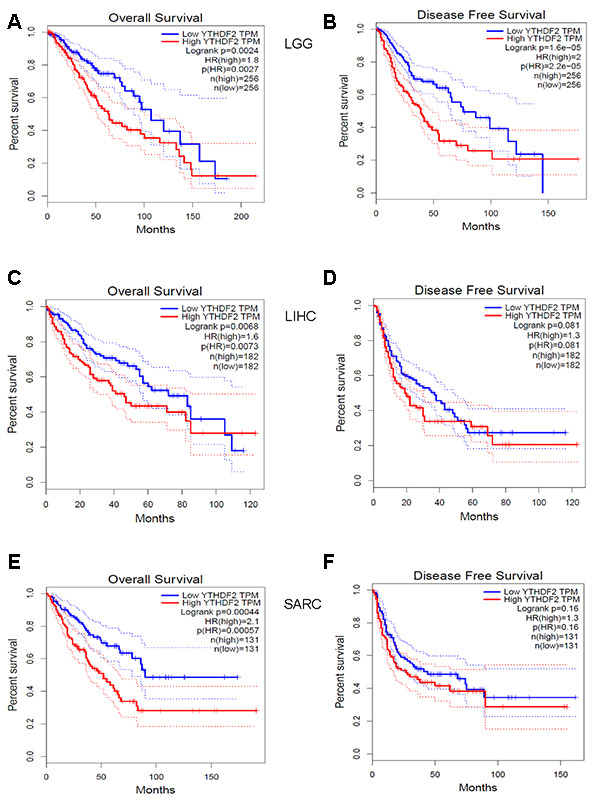
****Kaplan-Meier survival curves comparing YTHDF2 high and low expression (**A**, **B**) in LGG, (**C**, **D**) LIHC, and (**E**, **F**) SARC in datasets from the GEPIA database. (**A**) OS and (**B**) DFS survival curves in LGG (n = 256). (C) OS and (D) DFS survival curves in LIHC (n = 182). (**E**) OS and (**F**) DFS survival curves in SARC (n = 131). DFS, disease-free survival; LGG, lower-grade glioma; LIHC, liver hepatocellular carcinoma; SARC, sarcoma; OS, overall survival.

To further predict the prognostic potential of YTHDF2 in cancers, four databases (GEPIA, TIMER, OncoLnc, and Kaplan-Meier plotter) were used to evaluate the prognostic value of YTHDF2. The detailed results are summarized in Supplementary Table 3. In the GEPIA database, high YTHDF2 expression was associated with poorer overall survival (OS) and disease-free survival (DFS) in KICH (OS hazard ratio [HR] = 9.2, P= 0.011; DFS HR = 4.7, P = 0.031) and LGG (OS HR = 1.8, P = 0.0024; DFS HR = 2, P = 1.60e-05) (Figure 2A and 2B), whereas it was associated with better prognosis in KIRC (OS HR = 0.63, P = 0.0035; DFS HR = 0.63, P = 0.012). In addition, high YTHDF2 expression was associated with poorer OS but not poorer DFS in LIHC (OS HR = 1.6, P = 0.0068; DFS HR = 1.3, P = 0.081) (Figure 2C and 2D) and SARC (OS HR = 2.1, P = 0.00044; DFS HR = 1.3, P = 0.16) (Figure 2E and 2F), whereas it was associated with superior OS but not superior DFS in UCEC (OS HR = 0.48, P = 0.045; DFS HR = 0.63, P = 1.6). In the TIMER database, higher YTHDF2 expression was associated with poor OS in KICH (HR = 24.208, 95% confidence interval [CI] = 2.122-276.177, P = 0.01), LGG (HR = 2.749, 95% CI = 1.697-4.453, P = 0), LIHC (HR = 2.194, 95% CI =1.334-3.608, P = 0.002), and SARC (HR = 3.024, 95% CI = 1.725-5.302, P = 0). In the OncoLnc database, high YTHDF2 expression was associated with poor prognosis in LGG (Cox coefficient = 0.329, P = 0.00038), LIHC (Cox coefficient = 0.316, P = 0.00088) and SARC (Cox coefficient = 0.428, P = 0.00012), whereas it was associated with superior prognosis in READ (Cox coefficient = –0.53, P = 0.022). In the Kaplan-Meier plotter database, high YTHDF2 expression was associated with poor OS in LIHC (HR = 2.71, 95% CI = 1.9-3.87, P = 1.00e-08) and SARC (HR = 2.71, 95% CI = 1.62-4.55, P = 8.20e-05), whereas it was associated with superior OS in BLCA (HR = 0.69, 95% CI = 0.51-0.92, P = 0.011), KIRC (HR = 0.58, 95% CI = 0.43-0.78, P = 0.00029), LUAD (HR = 0.67, 95% CI = 0.5-0.9, P = 0.0078), OV (HR = 0.73, 95% CI = 0.56-0.95, P = 0.021), READ (HR = 0.47, 95% CI = 0.22-1.01, P = 0.048), and THYM (HR = 0, 95% CI = 0-inf, P = 0.038). These results suggest that YTHDF2 is a potential prognostic biomarker of LGG, LIHC, and SARC, and indicate the prognostic value of YTHDF2 expression may depend on cancer type.

We then used the “survival” TIMER module to confirm the prognostic value of YTHDF2 expression in LGG, LIHC, and SARC (Table 1). We explored the clinical impact of YTHDF2 and corrected for potential confounding factors with a multivariable Cox proportional hazard model. In the univariate analysis, YTHDF2, patient age, and all TIICs (B cells, CD4+ T cells, CD8+ T cells, macrophages, neutrophils. and DCs) had a significant impact on OS in LGG. YTHDF2, macrophages, and neutrophils had a significant impact on OS in LIHC, whereas YTHDF2, patient age, and CD4+ T cells had a significant impact on OS in SARC. In the multivariate analysis, we observed significant associations of YTHDF2, patient age, and macrophages with OS in LGG. However, only YTHDF2 was associated with OS in LIHC. In addition, associations between YTHDF2, patient age, CD4+ T cells, and OS were observed in SARC. By using the UALCAN database, higher YTHDF2 expression was associated with poor OS in LGG, LIHC, and SARC. YTHDF2 expression also impacted the OS in LGG, LIHC, and SARC with different clinicopathological parameters, such as gender and tumor grade (Supplementary Figure 1 and Supplementary Table 4). Although YTHDF2 expression was not significantly higher in LGG compared with normal samples (Supplementary Figure 2A), we found that YTHDF2 expression was higher in astrocytoma than in oligoastrocytoma and oligodendroglioma. YTHDF2 expression was higher in grade 3 LGG than in grade 2. In addition, higher YTHDF2 expression was associated with poor OS in all LGG and LGG with astrocytoma, but not oligoastrocytoma and oligodendroglioma (Supplementary Figure 2 and Supplementary Table 4).

### YTHDF2 expression is correlated with the immune infiltration level in LGG

As stated previously, some TIICs were independent predictors of survival in cancers (Table 1). Therefore, we investigated the correlation of YTHDF2 expression with immune infiltration levels in 32 cancer types from the TIMER database. The analysis showed that YTHDF2 expression was associated with tumor purity in 14 cancer types and B cell infiltration levels in 10 cancer types. In addition, YTHDF2 expression was associated with CD8+ T cell levels in 12 cancer types, CD4+T cell levels in 14 cancer types, macrophage levels in 14 cancer types, neutrophil levels in 12 cancer types, and DC levels in 12 cancer types (Supplementary Table 5).

**Figure 3:**
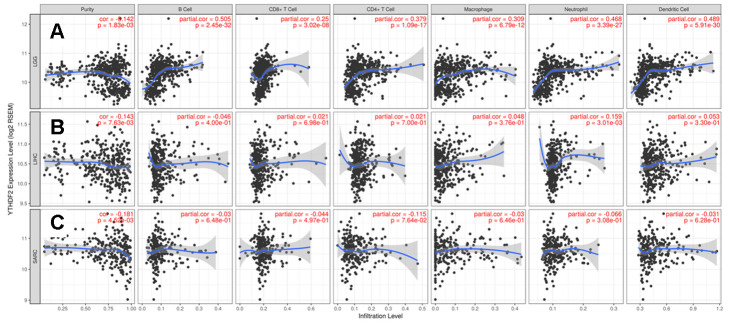
****Correlation of YTHDF2 expression with immune infiltration level in (**A**) LGG, (**B**) LIHC, and (**C**) SARC. LGG, lower-grade glioma; LIHC, liver hepatocellular carcinoma; SARC, sarcoma.

YTHDF2 expression was positively correlated with the levels of infiltrating B cells (r = 0.505, P = 2.45e-32), CD8+ T cells (r = 0.25, P = 3.02e-08), CD4+ T cells (r = 0.379, P = 1.09e-17), macrophages (r = 0.309, P = 6.79e-12), neutrophils (r = 0.468, P = 3.39e-27), and DCs (r = 0.489, P = 5.91e-30) in LGG (Figure 3A). However, YTHDF2 expression was only associated with neutrophils in LIHC (r = 0.159, P = 3.01e-03) (Figure 3B), and YTHDF2 expression had no significant correlations with infiltrating immune cell levels in SARC (Figure 3C). These findings strongly indicate that YTHDF2 plays an important role in immune infiltration in LGG.

### Correlation analysis between YTHDF2 expression and immune markers

To better understand the relationship between YTHDF2 and various infiltrating immune cells, we analyzed the correlations between YTHDF2 expression and the marker genes of different immune cells and functional T cells in LGG, LIHC, and SARC with the TIMER database. Table 2 shows YTHDF2 expression was associated with most marker genes of the various immune and T cells in LGG. However, YTHDF2 expression was associated with only 14 markers in LIHC and 13 markers in SARC (Table 2).

**Table 2:** Correlation between YTHDF2 and relate genes and markers of immune cells in TIMER.

**Description**	**Gene markers**	**LGG**				**LIHC**				**SARC**			
		**None**		**Purity**		**None**		**Purity**		**None**		**Purity**	
		**cor**	**p**	**cor**	**p**	**cor**	**p**	**cor**	**p**	**cor**	**p**	**cor**	**p**
CD8+T cell	CD8A	0.090	*	0.083	0.069	0.042	0.425	0.023	0.669	0.007	0.914	-0.001	0.983
	CD8B	-0.077	0.081	-0.070	0.128	0.069	0.186	0.047	0.384	-0.030	0.625	-0.041	0.523
T cell(general)	CD3D	0.158	***	0.159	***	0.065	0.214	0.054	0.315	-0.033	0.594	-0.039	0.545
	CD3E	0.174	***	0.181	***	-0.018	0.732	-0.026	0.624	-0.081	0.191	-0.088	0.171
	CD2	0.194	***	0.196	***	0.002	0.975	-0.012	0.829	-0.038	0.541	-0.045	0.478
B cell	CD19	0.222	***	0.221	***	0.080	0.125	0.085	0.116	-0.045	0.472	-0.030	0.635
	CD79A	0.273	***	0.304	***	-0.005	0.924	-0.016	0.773	0.016	0.802	0.031	0.632
Monocyte	CD86	0.436	***	0.442	***	0.122	*	0.107	*	0.075	0.231	0.067	0.295
	CSF1R	0.441	***	0.452	***	0.118	*	0.109	*	0.015	0.807	0.003	0.957
TAM	CCL2	0.222	***	0.214	***	0.099	0.057	0.103	0.056	-0.065	0.297	-0.074	0.246
	CD68	0.344	***	0.344	***	0.093	0.073	0.089	0.100	0.115	0.065	0.113	0.078
	IL10	0.305	***	0.307	***	0.136	**	0.139	*	0.150	*	0.140	*
M1 Macrophage	INOS (NOS2)	-0.024	0.582	-0.014	0.758	-0.122	*	-0.113	*	0.043	0.488	0.044	0.491
	IRF5	0.357	***	0.364	***	-0.149	**	-0.155	**	-0.040	0.523	-0.028	0.667
	COX2 (PTGS2)	0.113	*	0.111	*	0.099	0.057	0.100	0.062	-0.026	0.677	-0.028	0.659
M2 Macrophage	CD163	0.221	***	0.210	***	0.110	*	0.104	0.054	0.084	0.178	0.073	0.254
	VSIG4	0.426	***	0.420	***	0.145	**	0.139	*	0.091	0.145	0.078	0.222
	MS4A4A	0.296	***	0.302	***	0.121	*	0.112	*	0.053	0.396	0.036	0.576
Neutrophils	CD66b (CEACAM8)	0.100	*	0.097	*	0.072	0.169	0.083	0.122	0.064	0.300	0.071	0.266
	CD11b (ITGAM)	0.435	***	0.441	***	0.133	*	0.138	*	0.015	0.810	0.004	0.950
	CCR7	0.010	0.829	0.013	0.772	-0.010	0.855	-0.020	0.716	-0.170	**	-0.180	**
Natural killer cell	KIR2DL1	0.003	0.946	0.000	0.996	0.088	0.092	0.092	0.088	-0.025	0.688	-0.038	0.549
	KIR2DL3	0.085	0.055	0.086	0.059	0.078	0.132	0.082	0.130	-0.091	0.144	-0.106	0.097
	KIR2DL4	0.299	***	0.297	***	0.117	*	0.099	0.066	0.052	0.400	0.053	0.406
	KIR3DL1	-0.022	0.614	-0.029	0.520	0.067	0.195	0.059	0.273	-0.110	0.077	-0.134	*
	KIR3DL2	0.079	0.073	0.084	0.066	0.007	0.900	0.001	0.984	-0.037	0.555	-0.040	0.533
	KIR3DL3	0.028	0.523	0.031	0.498	0.079	0.130	0.082	0.127	-0.074	0.235	-0.078	0.223
	KIR2DS4	0.038	0.386	0.045	0.323	0.065	0.209	0.076	0.156	-0.037	0.551	-0.036	0.578
Dendritic cell	HLA-DPB1	0.304	***	0.312	***	0.045	0.389	0.029	0.591	-0.050	0.423	-0.059	0.359
	HLA-DQB1	0.258	***	0.258	***	0.051	0.324	0.031	0.565	-0.017	0.787	-0.025	0.695
	HLA-DRA	0.362	***	0.366	***	0.090	0.082	0.080	0.139	-0.012	0.846	-0.023	0.722
	HLA-DPA1	0.301	***	0.307	***	0.075	0.150	0.063	0.239	-0.063	0.309	-0.073	0.255
	BDCA-1(CD1C)	0.135	**	0.139	**	-0.067	0.198	-0.065	0.226	-0.238	***	-0.257	***
	BDCA-4(NRP1)	0.014	0.755	-0.003	0.948	0.101	0.051	0.109	*	0.135	*	0.127	*
	CD11c (ITGAX)	0.243	***	0.242	***	0.081	0.120	0.079	0.140	0.056	0.365	0.052	0.418
Th1	T-bet (TBX21)	0.121	**	0.112	*	0.053	0.306	0.046	0.390	-0.073	0.238	-0.082	0.202
	STAT4	-0.109	*	-0.103	*	-0.011	0.838	-0.022	0.679	0.005	0.941	-0.001	0.991
	STAT1	0.268	***	0.250	***	-0.010	0.841	-0.035	0.517	-0.148	*	-0.155	*
	IFN-γ (IFNG)	0.112	*	0.130	**	0.097	0.061	0.086	0.112	0.026	0.681	0.025	0.693
	TNF-α (TNF)	0.166	***	0.175	***	0.116	*	0.120	*	0.054	0.385	0.031	0.628
Th2	GATA3	0.276	***	0.272	***	0.032	0.534	0.023	0.663	0.134	*	0.154	*
	STAT6	0.207	***	0.222	***	0.013	0.801	0.015	0.780	-0.350	***	-0.346	***
	STAT5A	0.367	***	0.360	***	0.028	0.587	0.006	0.909	-0.183	**	-0.173	**
	IL13	0.047	0.283	0.045	0.322	0.027	0.607	0.032	0.548	0.039	0.533	0.064	0.321
Tfh	BCL6	0.120	**	0.118	*	0.172	**	0.194	***	-0.164	**	-0.186	**
	IL21	0.100	*	0.089	0.053	0.063	0.223	0.057	0.289	-0.002	0.969	-0.009	0.885
Th17	STAT3	0.290	***	0.265	***	0.178	**	0.184	**	-0.248	***	-0.269	***
	IL17A	-0.030	0.497	-0.027	0.551	0.011	0.833	0.035	0.511	0.017	0.784	0.037	0.561
Treg	FOXP3	-0.322	***	-0.317	***	0.040	0.441	0.052	0.336	0.126	*	0.125	0.051
	TGFβ (TGFB1)	0.415	***	0.419	***	0.004	0.934	0.008	0.882	0.160	*	0.149	*
	CCR8	0.028	0.531	0.020	0.662	0.070	0.181	0.075	0.166	0.064	0.305	0.050	0.436
STAT5B	-0.021	0.640	-0.030	0.512	-0.205	***	-0.201	***	-0.459	***	-0.474	***
T cell exhaustion	PD-1 (PDCD1)	0.188	***	0.176	***	0.016	0.756	0.007	0.896	0.114	0.067	0.123	0.054
	CTLA4	0.167	***	0.175	***	0.048	0.355	0.031	0.562	0.010	0.878	0.012	0.849
	LAG3	0.187	***	0.189	***	0.032	0.536	0.009	0.861	0.103	0.097	0.113	0.078
	TIM-3 (HAVCR2)	0.458	***	0.457	***	0.162	**	0.153	**	0.106	0.090	0.100	0.118
	GZMB	0.034	0.442	0.036	0.430	0.039	0.451	0.025	0.637	0.123	*	0.119	0.063

LGG, Brain Lower Grade Glioma; LIHC, Liver hepatocellular carcinoma; SARC, Sarcoma; TAM, tumor-associated macrophage; Th, T helper cell; Tfh, Follicular helper T cell;Treg, regulatory T cell; Cor, R value of Spearman’s correlation; None, correlation without adjustment. Purity, correlation adjusted by purity. P-value Significant Codes: 0 ≤ *** < 0.001 ≤ ** < 0.01 ≤ * < 0.05.

Interestingly, YTHDF2 expression was associated with gene markers of B cells, monocytes, TAMs, M2 macrophages, DCs, and Th2 cells in LGG (Table 2). These findings indicate that YTHDF2 may play a specific role in the regulation of macrophage polarization in LGG. We further investigated the relationship between YTHDF2 and the related genes and markers of TAMs. This analysis showed that YTHDF2 expression was related to TAM-related genes and markers, such as CCL2, CSF1, CSF1R, EGF, STAT3, STAT6, IL-6, IL-10, TLR4, TGFβ (TGFB1), LOX, PD-L1 (CD274), PD-L2 (PDCD1LG2), CD80, CD86, and MFGE8 (Table 3). Poor prognosis in LGG correlate with most TAM markers, including EGF, STAT3, STAT6, IL-6, IL-10, TGFβ (TGFB1), LOX, PD-L1 (CD274), PD-L2 (PDCD1LG2), CD80, and CD86 (Supplementary Table 6). These results further reveal that YTHDF2 has a strong relationship with TAM infiltration. We also found a significant relationship between YTHDF2 and DC markers, such as HLA-DPB1, HLA-DQB1, HLA-DRA, HLA-DPA1, BDCA-1(CD1C), and CD11c (ITGAX). In addition, a significant correlation between YTHDF2 and TGFβ (TGFB1) was observed in Treg cells, whereas TIM-3 (HAVCR2) correlate with T cell exhaustion (Table 2)., These results further suggest that YTHDF2 plays a role in immune escape in the LGG microenvironment.

**Table 3:** Correlation analysis between YTHDF2 and relate genes and markers of TAMs in TIMER.

**Description**	**Gene markers**	**LGG**				**LIHC**				**SARC**			
		**None**		**Purity**		**None**		**Purity**		**None**		**Purity**	
		**cor**	**p**	**cor**	**p**	**cor**	**p**	**cor**	**p**	**cor**	**p**	**cor**	**p**
TAMs	CCL2	0.222	***	0.214	***	0.099	0.057	0.103	0.056	-0.065	0.297	-0.074	0.246
	CSF1	0.399	***	0.389	***	0.223	***	0.257	***	-0.010	0.874	-0.033	0.604
	CSF1R	0.441	***	0.452	***	0.118	*	0.109	*	0.015	0.807	0.003	0.957
	EGF	0.280	***	0.293	***	0.356	***	0.361	***	-0.185	**	-0.201	**
	STAT3	0.290	***	0.265	***	0.178	***	0.184	***	-0.248	***	-0.269	***
	STAT6	0.207	***	0.222	***	0.013	0.801	0.015	0.780	-0.350	***	-0.346	***
	IL6	0.318	***	0.303	***	0.028	0.593	0.037	0.490	0.056	0.369	0.038	0.554
	IL10	0.305	***	0.307	***	0.136	**	0.139	**	0.150	*	0.140	*
	TLR4	0.114	**	0.116	*	0.174	***	0.176	**	-0.092	0.138	-0.104	0.104
	TGFβ (TGFB1)	0.415	***	0.419	***	0.004	0.934	0.008	0.882	0.160	**	0.149	*
	LOX	0.478	***	0.466	***	0.090	0.083	0.111	*	0.191	**	0.187	**
	PD-L1(CD274)	0.198	***	0.189	***	0.192	***	0.195	***	-0.110	0.076	-0.123	0.054
	PD-L2(PDCD1LG2)	0.454	***	0.456	***	0.104	*	0.096	0.075	-0.014	0.823	-0.026	0.687
	CD80	0.300	***	0.277	***	0.153	**	0.154	**	0.121	0.052	0.117	0.069
	CD86	0.436	***	0.442	***	0.122	*	0.107	*	0.075	0.231	0.067	0.295
	MFGE8	-0.366	***	-0.383	***	0.031	0.552	0.025	0.638	-0.433	***	-0.442	***

LGG, Brain Lower Grade Glioma; LIHC, Liver hepatocellular carcinoma; SARC, Sarcoma; TAMs, tumor-associated macrophages; Cor, R value of Spearman’s correlation; None, correlation without adjustment. Purity, correlation adjusted by purity. P-value Significant Codes: 0 ≤ *** < 0.001 ≤ ** < 0.01 ≤ * < 0.05.

### YTHDF2 expression is correlated with IDH1 level in LGG

**Figure 4:**
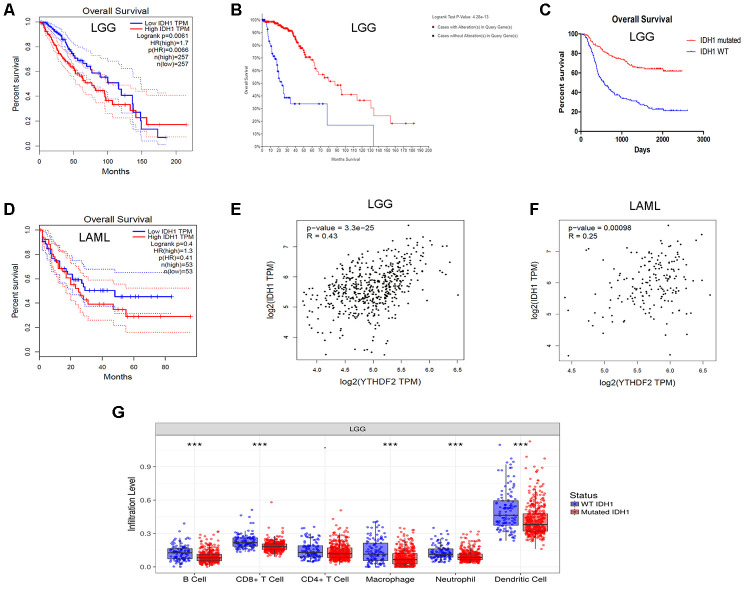
**Correlation of YTHDF2 expression with IDH1 level in LGG.** (**A**) High IDH1 expression was correlated with poor OS in the LGG GEPIA dataset. (**B**) LGG patients with IDH1 mutations had superior OS in the dataset from cBioPortal for Cancer Genomics. (**C**) IDH1 mutation led to a superior OS in gliomas. (**D**) IDH1 expression was not correlated with OS in the AML in GEPIA dataset. (**E**, **F**) YTHDF2 expression had a positive relationship with IDH1 in LGG and AML. (**G**) The immune infiltration levels were higher in IDH1-wild-type than in IDH1-mutant LGG. AML, acute myeloid leukemia; LGG, lower-grade glioma; OS, overall survival.

IDH1 mutations often occur in gliomas [47, 48] and AML [49, 50]. In addition, mutant IDH is highly associated with the regulation of the immune microenvironment in LGG [51]. Moreover, YTHDF2 is related to cancer stem cells (CSCs) in AML [52]. We attempted to find the relationship between YTHDF2 and IDH1 expression. We also analyzed the impact of the IDH1 mutation on immune infiltration levels in LGG. Interestingly, data from the GEPIA database showed that high IDH1 expression was associated with poor OS in LGG (HR = 1.7, P = 0.0061) (Figure 4A). LGG patients with IDH1 mutations had a superior OS according to the cBioPortal for Cancer Genomics analysis (Figure 4B). Chinese Glioma Cooperative Group (CGGA) data also indicated that the IDH1 mutation led to a superior OS in glioma (Figure 4C). However, the IDH1 mutation had no impact on OS in AML (Figure 4D). In addition, YTHDF2 expression has a moderate positive relationship with IDH1 in LGG (Figure 4E) and a weak positive relationship with IDH1 in AML (Figure 4F). YTHDF2 expression was weakly related to TAM-related genes and markers in AML (Supplementary Table 7). More importantly, the levels of infiltration B cells, CD8+ T cells, macrophages, neutrophils, and DCs were higher in IDH1-wild-type LGG than IDH1-mutant LGG (Figure 4G). These results suggest that YTHDF2 may play an important role in immune infiltration in LGG, especially IDH1-wild-type LGG, but not in AML.

### Enrichment analysis of YTHDF2 functional networks in LGG

**Figure 5:**
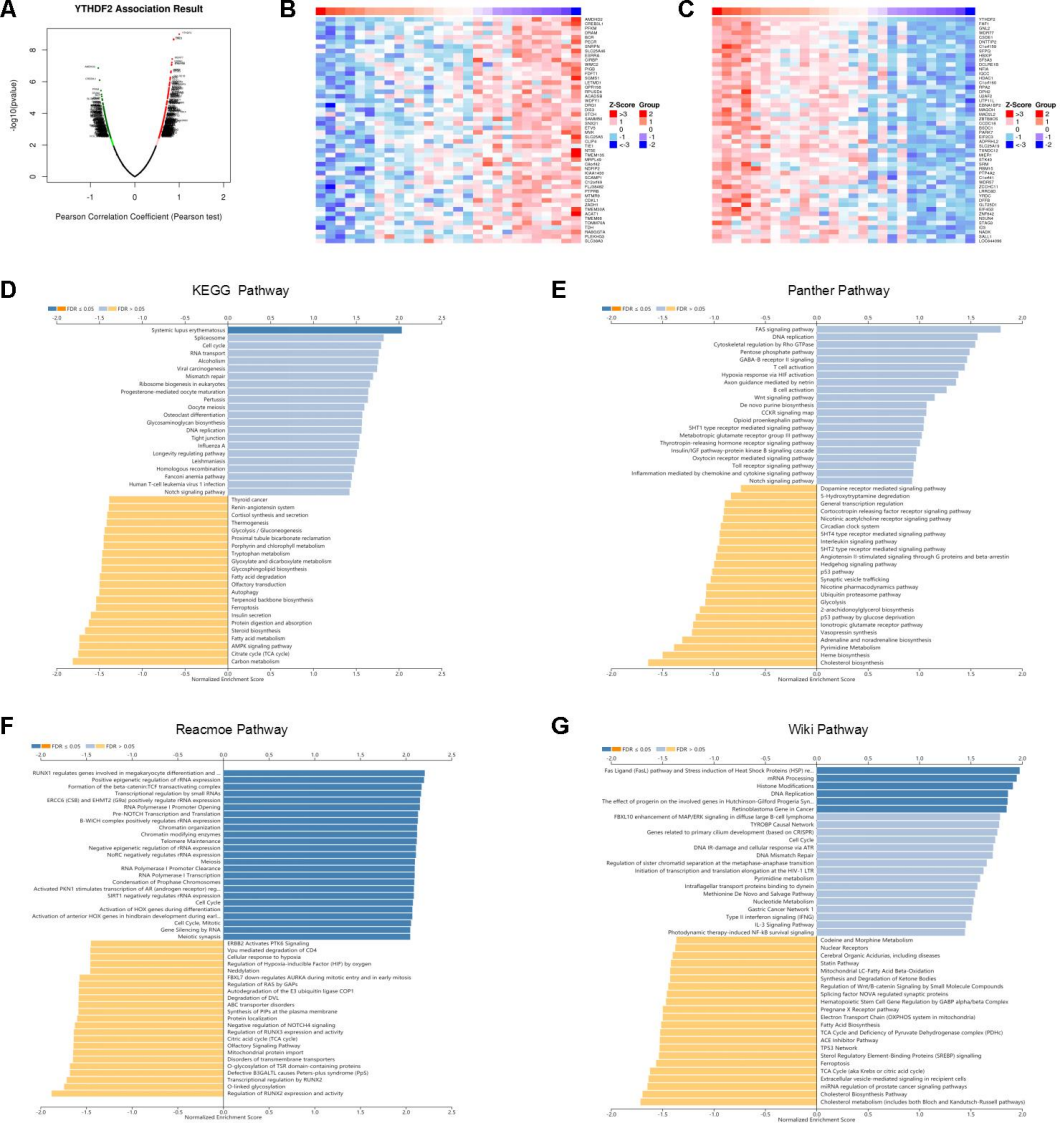
**Enrichment analysis of YTHDF2 functional networks in LGG by LinkedOmics.** (**A**) Volcano plot of genes differentially expressed in correlation with YTHDF2. (**B**, **C**) Heat maps of genes positively and negatively correlated with YTHDF2 (top 50). (**D**) KEGG pathway analysis of YTHDF2 by GSEA. (**E**) Panther pathway analysis of YTHDF2 by GSEA. (**F**) Reacmoe pathway analysis of YTHDF2 by GSEA. (**G**) Wiki pathway analysis of YTHDF2 by GSEA.

We used the LinkedOmics database to analyze YTHDF2 mRNA sequencing data from 27 LGG patients. The volcano plot in Figure 5A shows that YTHDF2 was positively correlated with 241 genes (dark-red dots) but negatively correlated with 195 genes (dark-green dots) (FDR< 0.05). The 50 significant gene sets positively and negatively associated with YTHDF2 are shown in the heat map (Figure 5B and 5C). The LinkedOmics GESA tool was used to perform the Gene Ontology and pathway enrichment analyses (Supplementary Table 8 and Figure 5D–5G). Supplementary Table 8 shows that in general the genes correlated with YTHDF2 were enriched in biological processes (double-strand break repair, DNA replication, cell cycle checkpoint, and mitotic cell cycle phase transition), cellular components (DNA packaging complex, protein-DNA complex, nuclear speck, replication fork, and chromosomal region), and molecular function (RNA polymerase II transcription factor binding, repressing transcription factor binding, NF-kappaB binding, nucleosome binding, and alcohol binding). Our results, demonstrating enrichment analyses for the KEGG, Panther, Reactome, and Wiki pathways, show the genes correlated with YTHDF2 were more enriched in cell cycle, TCA cycle, DNA replication, and the FAS signaling pathway.

## DISCUSSION

In the present study, we first performed a pan-cancer analysis to analyze YTHDF2 expression and prognostic value. Comprehensive analysis suggested that the differences in YTHDF2 expression and prognostic values in different types of cancer may reflect underlying mechanisms associated with different biological characteristics. Importantly, multivariate analysis confirmed that high YTHDF2 expression was an independent prognostic factor in patients with LGG, LIHC, or SARC. We found that YTHDF2 expression was higher in LGG compared with normal samples, although the difference was not significant. LGG are a diverse group of primary brain tumors, which mainly include astrocytoma, oligoastrocytoma, and oligodendroglioma. Previous studies have shown that astrocytic tumor type (vs. oligodendroglioma or oligo-dominant) was a poor prognostic indicator in patients with LGG [53–55]. We also found that YTHDF2 expression was higher in astrocytoma than in the other tumor types (oligoastrocytoma and oligodendroglioma). Moreover, high YTHDF2 expression was a prognostic factor in LGG with astrocytoma but not with oligoastrocytoma and oligodendroglioma. Similarly, the expression of YTHDF2 was higher in grade 3 LGG than in grade 2, and high YTHDF2 expression was a prognostic factor in LGG with different tumor grades. These results implied that YTHDF2 was a prognostic factor in LGG, especially with the more malignant subtype or higher tumor grade. However, more research is needed to verify the findings.

A second important finding from this study is that YTHDF2 expression positively correlated with the levels of infiltrating B cells, CD8+ T cells, CD4+ T cells, macrophages, neutrophils, and DCs in LGG. Notably, an association was found between YTHDF2 expression and TAM markers, such as CCL2, CSF1, CSF1R, EGF, STAT3, STAT6, IL-6, IL-10, TLR4, TGFβ (TGFB1), LOX, PD-L1 (CD274), PD-L2 (PDCD1LG2), CD80, CD86, and MFGE8. TAMs play a special role in regulating different steps of tumor progression and metastasis [56]. In glioma, CSCs can induce M2 macrophages, which secrete many cytokines, including TGF-β1 and IL-10, and facilitate immunosuppression [57]. Secretion of IL-10 and TGF-β was shown to facilitate an immunosuppressive microenvironment by inhibiting T cell proliferation in oral squamous cell carcinoma [58]. Interestingly, colony-stimulating factor-1 (CSF1) secreted from tumor cells was shown to induce macrophages to produce epidermal growth factor (EGF), which in turn promoted the migration of cancer cells [59]. In addition, inhibition of colony-stimulating factor-1 receptor (CSF1R) in TAMs suppressed the metastasis of pancreatic tumors [60]. The role of TAMs in immunosuppression has been widely studied. For instance, activation of the PD-1/PD-L1/PD-L2 and CTLA4/CD80/CD86 pathways leads to inhibition of TCR signal and T cell cytotoxic functions [61, 62]. Previously, it has been suggested that TAMs are attractive therapeutic targets, based on their important role in the tumor immunosuppressive microenvironment in cancer patients [56]. Another interesting finding is the association between YTHDF2 expression and DCs, Treg cells, and T cell exhaustion markers, such as HLA-DPB1, HLA-DQB1, HLA-DRA, HLA-DPA1, TGFβ, and TIM-3. Notably, TIM-3 is a crucial T cell exhaustion regulator [63]. DCs can promote tumor metastasis by increasing Treg cells and reducing CD8+T cell cytotoxicity [64]. In addition, some markers (tumor mutational burden [TMB], PD-1, and PD-L1) have been identified as the effectors of immunotherapy. TMB can be used as a biomarker to identify pediatric glioblastoma (GBM) patients who may benefit from immunotherapy [65]. However, another study found that high TMB is only found in 3.5% of GBM patients, and that IDH1-mutant gliomas are not enriched for high TMB [66]. PD-1 (PDCD1) promoter methylation is a prognostic factor in patients with LGG harboring IDH mutations [20]. A previous study found that PD-L2 expression upregulated in higher grade glioma and IDH-wild-type glioma. High PD-L2 expression was associated with poor survival in GBM [67]. Importantly, several immunotherapies have been evaluated in patients with glioma, including peptide vaccines, DC vaccines, oncolytic viruses, CAR-T cells, and checkpoint inhibitor therapy [68–70]. However, a previous study reported the response rates were low in refractory high-grade gliomas treated with PD-1 inhibitors [71]. TIGIT and PD-1 dual checkpoint blockade enhances antitumor immunity and survival in a murine GBM model [72]. Blocking PD-1/PD-L1 interactions together with MLN4924 therapy is a potential strategy for glioma treatment [73]. Gliomas treated with DC vaccination ± murine anti–PD-1 monoclonal antibody blockade or a colony-stimulating factor 1 receptor inhibitor (PLX3397) had prolonged survival in vivo [74]. Previous studies indicate that combination therapy with immune checkpoint blockade is effective for the treatment of malignant tumors, including GBM [75, 76].

Our third important finding is that YTHDF2 expression correlated with IDH1 expression in LGG. The analysis showed that high IDH1 expression was associated with poor OS in LGG. IDH1 mutations were associated with a superior OS. This is consistent with previous studies showing that IDH1 mutation is an independent favorable prognostic marker in glioma [47, 48]. In addition, the immune infiltration levels were higher in IDH1-wild-type LGG than in IDH1-mutant LGG. We showed that significant infiltration of immune cells, such as B cells, CD8+ T cells, CD4+ T cells, macrophages, neutrophils, and DCs, was linked to poor prognosis in LGG. In a previous study, IDH-wild-type gliomas exhibit a more prominent tumor infiltrating lymphocytes than IDH-mutant cases [77]. IDH1 mutations in gliomas caused leukocyte chemotaxis downregulation, resulting in suppression of the tumor-associated immune system [78]. As previously noted, IDH-mutant gliomas can escape the immune surveillance of natural killer cells [79]. More importantly, YTHDF2 expression has a positive relationship with IDH1 level. These results indicate that the role of YTHDF2 in immune infiltration in LGG may depend on IDH1 status. However, further investigations are needed to verify our findings.

Pathway enrichment analysis of YTHDF2 in LGG by GESA found that the genes correlated with YTHDF2 were more significantly enriched in cell cycle, TCA cycle, DNA replication, and the FAS signaling pathway. Interestingly, the most significant gene positively associated with YTHDF2, FAF1, can regulate antiviral immunity ([80, 81]). Moreover, notch family genes (the pathway found in the enrichment analysis) were prognostic biomarkers and correlated with immune infiltrates in gastric cancer ([82]). Because bioinformatics analysis was performed based on TCGA or GEO datasets, further biological experiments are needed to validate future results.

In summary, our data provide a comprehensive bioinformatics analysis of YTHDF2 expression and prognostic value in human cancers. High YTHDF2 expression correlates with poor prognosis and increased immune infiltration levels (including infiltration of B cells, CD8+ T cells, CD4+ T cells, macrophages, neutrophils and DCs) in LGG. YTHDF2 expression positively correlated with expression of several immune cell markers, including exhausted T cell markers, PD-1, TIM-3, and CTLA-4 in LGG. In addition, YTHDF2 expression positively correlated with TAM gene markers in LGG. Interestingly, YTHDF2 expression positively correlated with IDH1 expression in LGG. These findings suggest that YTHDF2 is a potential prognostic biomarker and correlates with tumor immune cells infiltration in LGG.

## MATERIALS AND METHODS

### GEPIA database analysis

GEPIA (http://gepia.cancer-pku.cn/index.html) [2] is an interactive web server for analyzing the RNA sequencing expression data of 9,736 tumors and 8,587 normal samples from the TCGA and the GTEx projects using a standard processing pipeline. GEPIA was used to analyze YTHDF2 expression and associated survival values (including OS and DFS) of YTHDF2 in 33 different cancer types. Using the Spearman method, correlation between YTHDF2 and IDH1 was determined. YTHDF2 values were represented on the x-axis, and IDH1 values were represented on the y-axis.

### TIMER database analysis

The TIMER database (https://cistrome.shinyapps.io/timer/) [3], which includes 10,897 samples across 32 cancer types from TCGA, is a comprehensive resource for estimating the abundance of six types of infiltrating immune cells, including B cells, CD4+ T cells, CD8+ T cells, neutrophils, macrophages, and DCs. We analyzed YTHDF2 expression in different cancer types via different expression modules and the correlation of YTHDF2 expression with the abundance of immune infiltrates via the gene module. Partial correlations between variables, when considering tumor purity, are shown on the left-most panel of the figure or table [83]. In addition, relationships between YTHDF2 expression and publicly available gene markers of TIICs were explored via correlation modules [84]. The Spearman method was used to determine the correlation coefficient.

### ONCOMINE analysis

ONCOMINE (http://www.oncomine.com) [4], an online cancer microarray database, was applied to analyze YTHDF2 mRNA levels in different cancers. The search filters were set as the following: differential analysis (cancer vs normal), cancer type (breast cancer), sample type (clinical specimen), data type (mRNA), and gene (YTHDF2). Thresholds were set as gene rank, 10%; fold change, 2; and P-value, 0.05.

### UALCAN database

UALCAN (http://ualcan.path.uab.edu/index.html) [5] is a portal for facilitating tumor subgroup gene expression and survival analyses. It was used to evaluate the mRNA levels and prognostic value of YTHDF2 in LGG patient and normal samples. A P value less than 0.05 was considered significant.

### PrognoScan database analysis

The PrognoScan database (http://www.abren.net/PrognoScan/) [6] was used to analyze the relationships between YTHDF2 expression and patient prognosis, such as OS and DFS, across publicly available cancer microarray datasets.

### Kaplan-Meier plotter database analysis

The Kaplan-Meier plotter (http://kmplot.com/analysis/) [7] is capable of assessing the effect of 54,675 genes on survival in 21 cancer types. The correlation between YTHDF2 expression and survival was analyzed by the pan-cancer module of the Kaplan-Meier plotter. The HR with 95% CI and the log-rank P-value were determined.

### OncoLnc database analysis

OncoLnc (http://www.oncolnc.org/) [8] is an interactive tool for exploring survival correlations, and for downloading clinical data coupled to expression data for mRNAs, miRNAs, and long noncoding RNAs. The correlation between YTHDF2 expression and survival was analyzed by OncoLnc. The Cox correlation coefficient and P-value were calculated.

### CGGA database analysis

A total of 118 glioma samples (82 samples with IDH1 mutation and 37 with wild-type IDH1) from CGGA were analyzed to determine the association of IDH1 with survival [9]. GraphPad Prism software was used to generate a survival curve, and the log-rank test was used to assess the statistical significance.

### cBioportal for Cancer Genomics database analysis

The cBioportal Cancer Genomics database (https://www.cbioportal.org) [10], which was originally developed at Memorial Sloan Kettering Cancer Center, enables users to visualize, analyze, and download large-scale cancer genomics datasets. The survival associated with IDH1 alterations in LGG was analyzed, and the log-rank test P-value was calculated. Determination of the correlation between YTHDF2 and IDH1 was performed using the Spearman and Pearson methods.

### LinkedOmics dataset

LinkedOmics (http://www.linkedomics.org/login.php) [11] is a publicly available portal that includes multi-omics data from all 32 TCGA cancer types. It provides a unique platform for biologists and clinicians to access, analyze, and compare cancer multi-omics data within and across tumor types.

## Supplementary Material

Supplementary Figures

Supplementary Table 1

Supplementary Table 2

Supplementary Table 3

Supplementary Table 4

Supplementary Table 5

Supplementary Tables 6, 7 and 8
