# Supplementary material for: YTHDF2 correlates with tumor immune infiltrates in lower-grade glioma
Source: Aging (Albany NY). 2020 Sep 27;12(18):18476–500. doi: 10.18632/aging.103812 (PMC7585119; doi:10.18632/aging.103812)
Supplement: Supplementary Figures [file aging-12-103812-s007..pdf]

SUPPLEMENTARY FIGURES

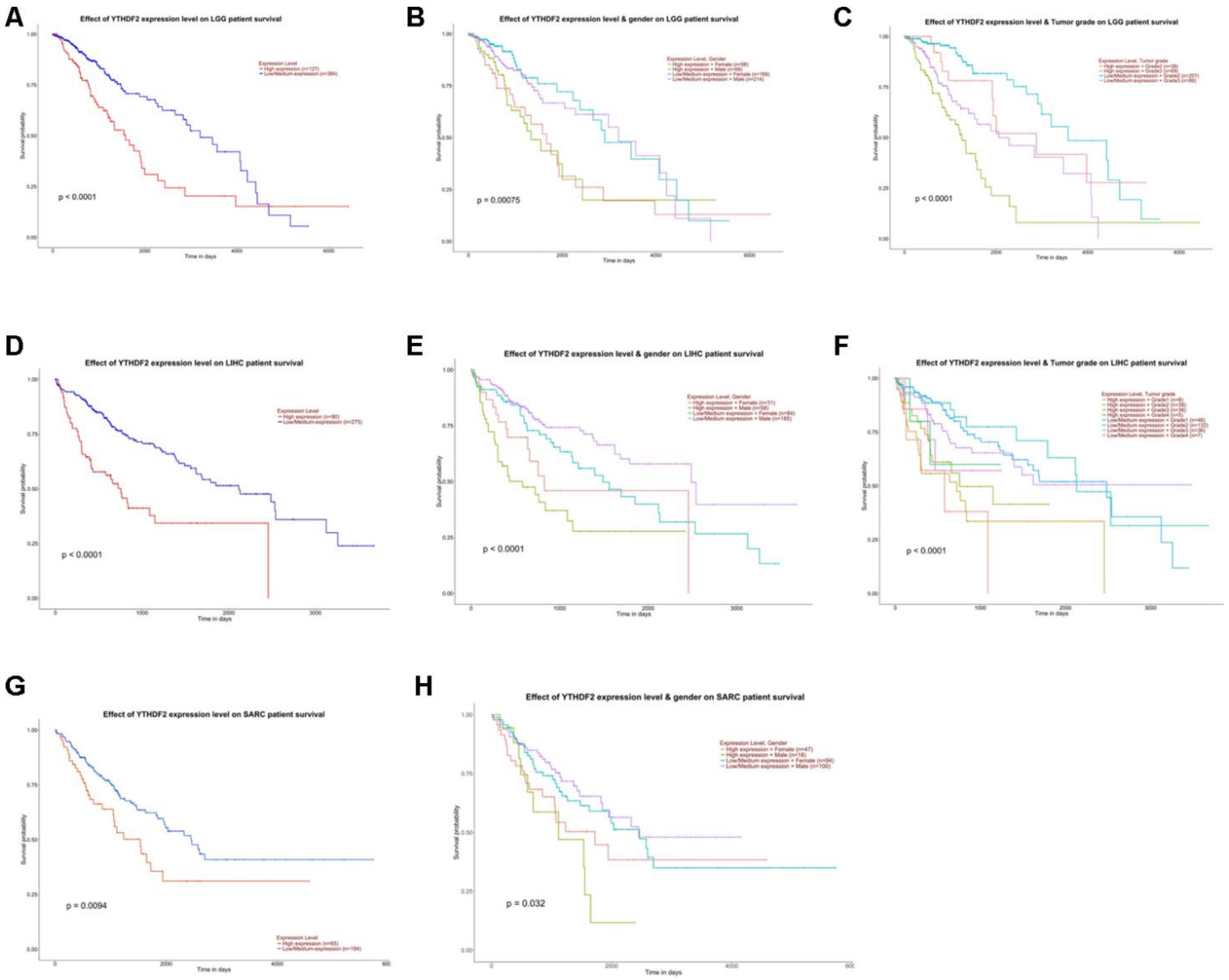

Supplementary Figure 1. Prognostic YTHDF2 values in cancers analyzed by the UALCAN database. (**A**) LGG. (**B**) LGG with different gender. (**C**) LGG with different tumor grade. (**D**) LIHC. (**E**) LIHC with different gender. (**F**) LIHC with different tumor grade. (**G**) SARC. (**H**) SARC with different gender. LGG, lower-grade glioma; LIHC, liver hepatocellular carcinoma; SARC, sarcoma.

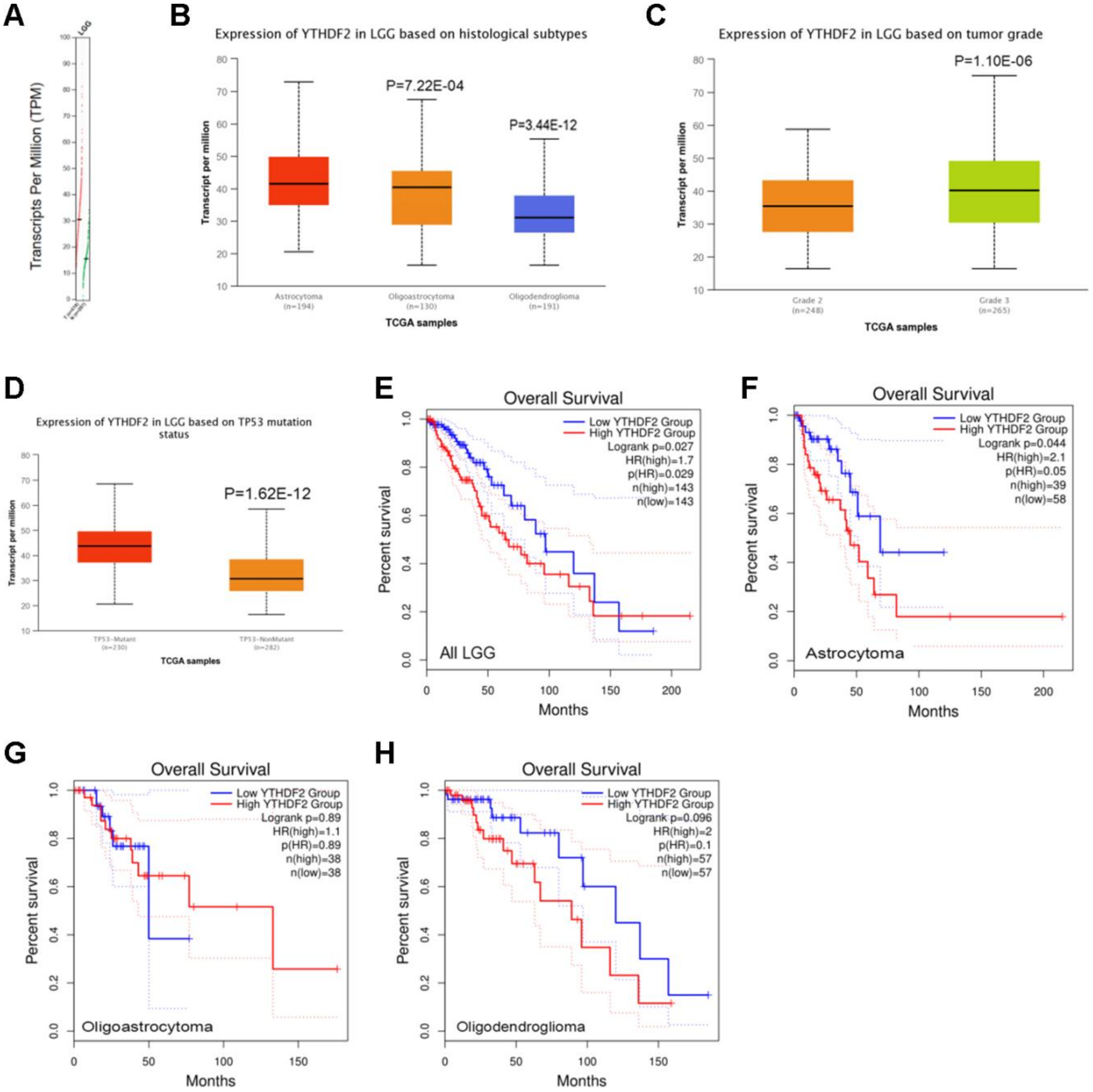

Supplementary Figure 2. Expression and overall survival of YTHDF2 in LGG analyzed by the UALCAN and GEPIA databases. (**A**) Expression level of YTHDF2 in LGG compared with normal sample. (**B**) Expression level of YTHDF2 in LGG based on histological subtypes. (**C**) Expression level of YTHDF2 in LGG based on tumor grade. (**D**) Expression level of YTHDF2 in LGG based on TP53 mutation status. (**E**) Overall survival of YTHDF2 in all LGG patients. (**F**) Overall survival of YTHDF2 in LGG patients with astrocytoma. (**G**) Overall survival of YTHDF2 in LGG patients with oligoastrocytoma. (**H**) Overall survival of YTHDF2 in LGG patients with oligodendroglioma. LGG, lower-grade glioma.
