# Supplementary material for: YTHDF2 correlates with tumor immune infiltrates in lower-grade glioma
Source: Aging (Albany NY). 2020 Sep 27;12(18):18476–500. doi: 10.18632/aging.103812 (PMC7585119; doi:10.18632/aging.103812)
Supplement: Supplementary Table 1 [file aging-12-103812-s001..pdf]

Supplementary Table 1. The significant Datasets of the YTHDF2 in Human Cancers (ONCOMINE database).

Cancer type	Sub cancer type	t-Test	Fold Change	P-value	Study
**Brain and CNS Cancer**	Anaplastic Oligoastrocytoma vs. Normal	5.874	2.433	1.90E-04	French Brain Statistics
	Glioblastoma vs. Normal	-16.491	-2.762	3.97E-13	Lee Brain Statistics
**Breast cancer**	Fibroadenoma vs. Normal	5.597	9.945	0.003	Sorlie Breast Statistics
	Fibroadenoma vs. Normal	6.235	11.69	0.002	Sorlie Breast 2 Statistics
**Cervical Cancer**	Cervical Cancer vs. Normal	6.66	2.061	3.58E-08	Pyeon Multi-cancer Statistics
**Head and Neck cancer**	Oral Cavity Carcinoma vs. Normal	5.523	2.838	1.78E-04	Pyeon Multi-cancer Statistics
**Kidney cancer**	Renal Wilms Tumor vs. Normal	4.817	2.3	0.002	Yusenko Renal Statistics
**Other cancer**	Parathyroid Hyperplasia vs. Normal	4.171	2.55	8.54E-04	Morrison Parathyroid Statistics
	Parathyroid Gland Adenoma vs. Normal	5.064	2.038	3.88E-04	Morrison Parathyroid Statistics
