# Supplementary material for: YTHDF2 correlates with tumor immune infiltrates in lower-grade glioma
Source: Aging (Albany NY). 2020 Sep 27;12(18):18476–500. doi: 10.18632/aging.103812 (PMC7585119; doi:10.18632/aging.103812)
Supplement: Supplementary Table 2 [file aging-12-103812-s004..doc]

| Supplementary Table 2. Prognostic values of YTHDF2 in cancers analyzed by PrognoScan database. | | | | | | | | | | |
| --- | --- | --- | --- | --- | --- | --- | --- | --- | --- | --- |
|
| **ID_NAME** | **DATASET** | **CANCER TYPE** | **SUBTYPE** | **ENDPOINT** | **COHORT** | **CONTRIBUTOR** | **PROBE ID** | **N** | **COX P-VALUE** | **HR [95% CI]** |
| YTHDF2 | GSE5287 | Bladder cancer |  | OS | Aarhus (1995-2004) | Als | 217812_at | 30 | 0.258 | 0.52 [0.17 - 1.61] |
| YTHDF2 | GSE13507 | Bladder cancer |  | OS | CNUH | Kim | ILMN_1730658 | 165 | 0.475 | 1.23 [0.70 - 2.15] |
| YTHDF2 | GSE13507 | Bladder cancer | Transitional cell carcinoma | DSS | CNUH | Kim | ILMN_1730658 | 165 | 0.412 | 1.44 [0.60 - 3.42] |
| YTHDF2 | GSE12417-GPL96 | Blood cancer | AML | OS | AMLCG (1999-2003) | Metzeler | 217812_at | 163 | 0.842 | 0.97 [0.70 - 1.34] |
| YTHDF2 | GSE12417-GPL97 | Blood cancer | AML | OS | AMLCG (1999-2003) | Metzeler | 222430_s_at | 163 | 0.367 | 1.15 [0.85 - 1.57] |
| YTHDF2 | GSE12417-GPL570 | Blood cancer | AML | OS | AMLCG (2004) | Metzeler | 222430_s_at | 79 | 0.508 | 1.20 [0.70 - 2.05] |
| YTHDF2 | GSE12417-GPL570 | Blood cancer | AML | OS | AMLCG (2004) | Metzeler | 217812_at | 79 | 0.370 | 1.40 [0.67 - 2.91] |
| YTHDF2 | GSE5122 | Blood cancer | AML | OS | San Diego | Raponi | 217812_at | 58 | 0.730 | 0.91 [0.54 - 1.54] |
| YTHDF2 | GSE8970 | Blood cancer | AML | OS | San Diego | Raponi | 217812_at | 34 | 0.067 | 2.47 [0.94 - 6.48] |
| YTHDF2 | GSE4475 | Blood cancer | B-cell lymphoma | OS | Berlin (2003-2005) | Hummel | 217812_at | 158 | 0.178 | 0.71 [0.43 - 1.17] |
| YTHDF2 | E-TABM-346 | Blood cancer | DLBCL | OS | GELA (1998-2000) | Jais | 217812_at | 53 | 0.684 | 0.73 [0.17 - 3.26] |
| YTHDF2 | E-TABM-346 | Blood cancer | DLBCL | EFS | GELA (1998-2000) | Jais | 217812_at | 53 | 0.709 | 1.32 [0.31 - 5.58] |
| YTHDF2 | GSE16131-GPL96 | Blood cancer | Follicular lymphoma | OS | NCI (1974-2001) | Dave | 217812_at | 180 | 0.744 | 0.86 [0.34 - 2.17] |
| YTHDF2 | GSE16131-GPL97 | Blood cancer | Follicular lymphoma | OS | NCI (1974-2001) | Dave | 222430_s_at | 180 | 0.182 | 0.61 [0.29 - 1.26] |
| YTHDF2 | GSE2658 | Blood cancer | Multiple myeloma | DSS | Arkansas | Zhan | 222430_s_at | 559 | 0.480 | 1.22 [0.70 - 2.11] |
| YTHDF2 | GSE2658 | Blood cancer | Multiple myeloma | DSS | Arkansas | Zhan | 217812_at | 559 | 0.704 | 1.18 [0.51 - 2.71] |
| YTHDF2 | GSE4271-GPL96 | Brain cancer | Astrocytoma | OS | MDA | Phillips | 217812_at | 77 | 0.003 | 2.51 [1.38 - 4.58] |
| YTHDF2 | GSE4271-GPL97 | Brain cancer | Astrocytoma | OS | MDA | Phillips | 222430_s_at | 77 | 0.022 | 2.42 [1.14 - 5.18] |
| YTHDF2 | GSE7696 | Brain cancer | Glioblastoma | OS | Lausanne | Murat | 222430_s_at | 70 | 0.833 | 0.91 [0.39 - 2.12] |
| YTHDF2 | GSE7696 | Brain cancer | Glioblastoma | OS | Lausanne | Murat | 217812_at | 70 | 0.449 | 0.77 [0.39 - 1.51] |
| YTHDF2 | GSE4412-GPL96 | Brain cancer | Glioma | OS | UCLA (1996-2003) | Freije | 217812_at | 74 | 0.095 | 2.85 [0.84 - 9.70] |
| YTHDF2 | GSE4412-GPL97 | Brain cancer | Glioma | OS | UCLA (1996-2003) | Freije | 222430_s_at | 74 | 0.125 | 2.02 [0.82 - 4.96] |
| YTHDF2 | GSE16581 | Brain cancer | Meningioma | OS | UCLA | Lee | 222430_s_at | 67 | 0.292 | 0.24 [0.02 - 3.39] |
| YTHDF2 | GSE16581 | Brain cancer | Meningioma | OS | UCLA | Lee | 217812_at | 67 | 0.376 | 0.29 [0.02 - 4.50] |
| YTHDF2 | GSE19615 | Breast cancer |  | DMFS | DF/HCC | Li | 222430_s_at | 115 | 0.598 | 0.58 [0.08 - 4.34] |
| YTHDF2 | GSE19615 | Breast cancer |  | DMFS | DF/HCC | Li | 217812_at | 115 | 0.414 | 0.28 [0.01 - 5.93] |
| YTHDF2 | GSE12276 | Breast cancer |  | RFS | EMC | Bos | 217812_at | 204 | 0.535 | 1.20 [0.68 - 2.10] |
| YTHDF2 | GSE12276 | Breast cancer |  | RFS | EMC | Bos | 222430_s_at | 204 | 0.389 | 0.82 [0.52 - 1.29] |
| YTHDF2 | GSE6532-GPL570 | Breast cancer |  | RFS | GUYT | Loi | 222430_s_at | 87 | 0.872 | 0.91 [0.29 - 2.83] |
| YTHDF2 | GSE6532-GPL570 | Breast cancer |  | DMFS | GUYT | Loi | 222430_s_at | 87 | 0.872 | 0.91 [0.29 - 2.83] |
| YTHDF2 | GSE6532-GPL570 | Breast cancer |  | RFS | GUYT | Loi | 217812_at | 87 | 0.636 | 0.70 [0.16 - 3.03] |
| YTHDF2 | GSE6532-GPL570 | Breast cancer |  | DMFS | GUYT | Loi | 217812_at | 87 | 0.636 | 0.70 [0.16 - 3.03] |
| YTHDF2 | GSE9195 | Breast cancer |  | DMFS | GUYT2 | Loi | 217812_at | 77 | 0.584 | 0.56 [0.07 - 4.50] |
| YTHDF2 | GSE9195 | Breast cancer |  | RFS | GUYT2 | Loi | 222430_s_at | 77 | 0.125 | 0.36 [0.10 - 1.33] |
| YTHDF2 | GSE9195 | Breast cancer |  | RFS | GUYT2 | Loi | 217812_at | 77 | 0.015 | 0.10 [0.02 - 0.64] |
| YTHDF2 | GSE9195 | Breast cancer |  | DMFS | GUYT2 | Loi | 222430_s_at | 77 | 0.683 | 0.74 [0.17 - 3.19] |
| YTHDF2 | GSE12093 | Breast cancer |  | DMFS | IO, NCI, TUM, CCF (1992-2000) | Zhang | 217812_at | 136 | 0.903 | 0.90 [0.16 - 4.95] |
| YTHDF2 | GSE11121 | Breast cancer |  | DMFS | Mainz (1988-1998) | Schmidt | 217812_at | 200 | 0.551 | 1.57 [0.35 - 7.00] |
| YTHDF2 | GSE1378 | Breast cancer |  | RFS | MGH (1987-2000) | Ma | 7450 | 60 | 0.852 | 0.90 [0.31 - 2.64] |
| YTHDF2 | GSE1379 | Breast cancer |  | RFS | MGH (1987-2000) | Ma | 7450 | 60 | 0.950 | 0.94 [0.16 - 5.72] |
| YTHDF2 | GSE2034 | Breast cancer |  | DMFS | Rotterdam (1980-1995) | Wang | 217812_at | 286 | 0.638 | 1.24 [0.51 - 3.03] |
| YTHDF2 | GSE1456-GPL96 | Breast cancer |  | OS | Stockholm (1994-1996) | Pawitan | 217812_at | 159 | 0.061 | 3.12 [0.95 - 10.24] |
| YTHDF2 | GSE1456-GPL96 | Breast cancer |  | RFS | Stockholm (1994-1996) | Pawitan | 217812_at | 159 | 0.308 | 1.90 [0.55 - 6.55] |
| YTHDF2 | GSE1456-GPL96 | Breast cancer |  | DSS | Stockholm (1994-1996) | Pawitan | 217812_at | 159 | 0.302 | 2.11 [0.51 - 8.73] |
| YTHDF2 | GSE1456-GPL97 | Breast cancer |  | OS | Stockholm (1994-1996) | Pawitan | 222430_s_at | 159 | 0.114 | 1.95 [0.85 - 4.47] |
| YTHDF2 | GSE1456-GPL97 | Breast cancer |  | RFS | Stockholm (1994-1996) | Pawitan | 222430_s_at | 159 | 0.033 | 2.48 [1.08 - 5.72] |
| YTHDF2 | GSE1456-GPL97 | Breast cancer |  | DSS | Stockholm (1994-1996) | Pawitan | 222430_s_at | 159 | 0.066 | 2.46 [0.94 - 6.39] |
| YTHDF2 | GSE7378 | Breast cancer |  | DFS | UCSF | Zhou | 217812_at | 54 | 0.155 | 2.81 [0.68 - 11.70] |
| YTHDF2 | E-TABM-158 | Breast cancer |  | OS | UCSF, CPMC (1989-1997) | Chin | 217812_at | 117 | 0.718 | 0.88 [0.43 - 1.78] |
| YTHDF2 | E-TABM-158 | Breast cancer |  | DMFS | UCSF, CPMC (1989-1997) | Chin | 217812_at | 117 | 0.131 | 0.52 [0.22 - 1.22] |
| YTHDF2 | E-TABM-158 | Breast cancer |  | RFS | UCSF, CPMC (1989-1997) | Chin | 217812_at | 117 | 0.718 | 0.88 [0.43 - 1.78] |
| YTHDF2 | E-TABM-158 | Breast cancer |  | DSS | UCSF, CPMC (1989-1997) | Chin | 217812_at | 117 | 0.524 | 0.76 [0.33 - 1.76] |
| YTHDF2 | GSE3494-GPL96 | Breast cancer |  | DSS | Uppsala (1987-1989) | Miller | 217812_at | 236 | 0.469 | 1.57 [0.46 - 5.30] |
| YTHDF2 | GSE3494-GPL97 | Breast cancer |  | DSS | Uppsala (1987-1989) | Miller | 222430_s_at | 236 | 0.190 | 2.10 [0.69 - 6.33] |
| YTHDF2 | GSE4922-GPL96 | Breast cancer |  | DFS | Uppsala (1987-1989) | Ivshina | 217812_at | 249 | 0.828 | 1.11 [0.43 - 2.85] |
| YTHDF2 | GSE4922-GPL97 | Breast cancer |  | DFS | Uppsala (1987-1989) | Ivshina | 222430_s_at | 249 | 0.709 | 1.18 [0.49 - 2.82] |
| YTHDF2 | GSE2990 | Breast cancer |  | DMFS | Uppsala, Oxford | Sotiriou | 217812_at | 125 | 0.139 | 1.75 [0.83 - 3.65] |
| YTHDF2 | GSE2990 | Breast cancer |  | RFS | Uppsala, Oxford | Sotiriou | 217812_at | 125 | 0.529 | 1.20 [0.67 - 2.15] |
| YTHDF2 | GSE2990 | Breast cancer |  | DMFS | Uppsala, Oxford | Sotiriou | 217812_at | 54 | 0.584 | 0.82 [0.41 - 1.66] |
| YTHDF2 | GSE2990 | Breast cancer |  | RFS | Uppsala, Oxford | Sotiriou | 217812_at | 62 | 0.496 | 0.82 [0.46 - 1.46] |
| YTHDF2 | GSE7390 | Breast cancer |  | RFS | Uppsala, Oxford, Stockholm, IGR, GUYT, CRH (1980-1998) | Desmedt | 217812_at | 198 | 0.145 | 0.64 [0.36 - 1.16] |
| YTHDF2 | GSE7390 | Breast cancer |  | DMFS | Uppsala, Oxford, Stockholm, IGR, GUYT, CRH (1980-1998) | Desmedt | 217812_at | 198 | 0.269 | 0.68 [0.34 - 1.35] |
| YTHDF2 | GSE7390 | Breast cancer |  | OS | Uppsala, Oxford, Stockholm, IGR, GUYT, CRH (1980-1998) | Desmedt | 217812_at | 198 | 0.198 | 0.62 [0.30 - 1.29] |
| YTHDF2 | GSE12945 | Colorectal cancer |  | DFS | Berlin | Staub | 217812_at | 51 | 0.545 | 1.68 [0.31 - 9.03] |
| YTHDF2 | GSE12945 | Colorectal cancer |  | OS | Berlin | Staub | 217812_at | 62 | 0.362 | 1.67 [0.56 - 4.99] |
| YTHDF2 | GSE17536 | Colorectal cancer |  | OS | MCC | Smith | 217812_at | 177 | 0.532 | 1.32 [0.55 - 3.19] |
| YTHDF2 | GSE17536 | Colorectal cancer |  | DFS | MCC | Smith | 222430_s_at | 145 | 0.429 | 0.67 [0.25 - 1.80] |
| YTHDF2 | GSE17536 | Colorectal cancer |  | DFS | MCC | Smith | 217812_at | 145 | 0.504 | 0.68 [0.22 - 2.11] |
| YTHDF2 | GSE17536 | Colorectal cancer |  | DSS | MCC | Smith | 217812_at | 177 | 0.541 | 1.38 [0.49 - 3.84] |
| YTHDF2 | GSE17536 | Colorectal cancer |  | OS | MCC | Smith | 222430_s_at | 177 | 0.867 | 1.06 [0.51 - 2.22] |
| YTHDF2 | GSE17536 | Colorectal cancer |  | DSS | MCC | Smith | 222430_s_at | 177 | 0.746 | 1.15 [0.49 - 2.71] |
| YTHDF2 | GSE14333 | Colorectal cancer |  | DFS | Melbourne | Jorissen | 222430_s_at | 226 | 0.201 | 0.64 [0.32 - 1.27] |
| YTHDF2 | GSE14333 | Colorectal cancer |  | DFS | Melbourne | Jorissen | 217812_at | 226 | 0.191 | 0.50 [0.18 - 1.41] |
| YTHDF2 | GSE17537 | Colorectal cancer |  | DFS | VMC | Smith | 217812_at | 55 | 0.334 | 2.18 [0.45 - 10.65] |
| YTHDF2 | GSE17537 | Colorectal cancer |  | DFS | VMC | Smith | 222430_s_at | 55 | 0.049 | 3.29 [1.01 - 10.72] |
| YTHDF2 | GSE17537 | Colorectal cancer |  | DFS | VMC | Smith | 217812_at | 49 | 0.433 | 2.33 [0.28 - 19.14] |
| YTHDF2 | GSE17537 | Colorectal cancer |  | DFS | VMC | Smith | 222430_s_at | 49 | 0.111 | 3.56 [0.75 - 16.96] |
| YTHDF2 | GSE17537 | Colorectal cancer |  | OS | VMC | Smith | 222430_s_at | 55 | 0.070 | 2.61 [0.92 - 7.40] |
| YTHDF2 | GSE17537 | Colorectal cancer |  | OS | VMC | Smith | 217812_at | 55 | 0.222 | 2.49 [0.58 - 10.80] |
| YTHDF2 | GSE22138 | Eye cancer | Uveal melanoma | DMFS | BRCIC | Laurent | 217812_at | 63 | 0.924 | 1.02 [0.69 - 1.49] |
| YTHDF2 | GSE22138 | Eye cancer | Uveal melanoma | DMFS | BRCIC | Laurent | 222430_s_at | 63 | 0.394 | 1.21 [0.78 - 1.88] |
| YTHDF2 | GSE2837 | Head and neck cancer | Squamous cell carcinoma | RFS | VUMC, VAMC, UTMDACC (1992-2005) | Chung | g7705410_3p_at | 28 | 0.351 | 1.26 [0.77 - 2.07] |
| YTHDF2 | GSE2837 | Head and neck cancer | Squamous cell carcinoma | RFS | VUMC, VAMC, UTMDACC (1992-2005) | Chung | g12803468_3p_a_at | 28 | 0.904 | 1.06 [0.40 - 2.84] |
| YTHDF2 | jacob-00182-CANDF | Lung cancer | Adenocarcinoma | OS | CAN/DF | Shedden | 217812_at | 82 | 0.072 | 0.37 [0.13 - 1.09] |
| YTHDF2 | jacob-00182-HLM | Lung cancer | Adenocarcinoma | OS | HLM | Shedden | 217812_at | 79 | 0.642 | 0.78 [0.28 - 2.20] |
| YTHDF2 | jacob-00182-MSK | Lung cancer | Adenocarcinoma | OS | MSK | Shedden | 217812_at | 104 | 0.063 | 0.32 [0.09 - 1.06] |
| YTHDF2 | GSE13213 | Lung cancer | Adenocarcinoma | OS | Nagoya (1995-1999, 2002-2004) | Tomida | A_32_P99275 | 117 | 0.190 | 0.61 [0.29 - 1.28] |
| YTHDF2 | GSE13213 | Lung cancer | Adenocarcinoma | OS | Nagoya (1995-1999, 2002-2004) | Tomida | A_23_P103414 | 117 | 0.499 | 0.76 [0.34 - 1.68] |
| YTHDF2 | GSE31210 | Lung cancer | Adenocarcinoma | OS | NCCRI | Okayama | 217812_at | 204 | 0.102 | 0.15 [0.01 - 1.47] |
| YTHDF2 | GSE31210 | Lung cancer | Adenocarcinoma | OS | NCCRI | Okayama | 222430_s_at | 204 | 0.494 | 1.72 [0.36 - 8.13] |
| YTHDF2 | GSE31210 | Lung cancer | Adenocarcinoma | RFS | NCCRI | Okayama | 217812_at | 204 | 0.072 | 0.21 [0.04 - 1.15] |
| YTHDF2 | GSE31210 | Lung cancer | Adenocarcinoma | RFS | NCCRI | Okayama | 222430_s_at | 204 | 0.302 | 1.83 [0.58 - 5.81] |
| YTHDF2 | jacob-00182-UM | Lung cancer | Adenocarcinoma | OS | UM | Shedden | 217812_at | 178 | 0.094 | 0.44 [0.17 - 1.15] |
| YTHDF2 | GSE11117 | Lung cancer | NSCLC | OS | Basel (2002-2005) | Baty | H200002956 | 41 | 0.209 | 1.53 [0.79 - 2.99] |
| YTHDF2 | GSE3141 | Lung cancer | NSCLC | OS | Duke | Bild | 217812_at | 111 | 0.101 | 2.09 [0.87 - 5.04] |
| YTHDF2 | GSE3141 | Lung cancer | NSCLC | OS | Duke | Bild | 222430_s_at | 111 | 0.169 | 1.70 [0.80 - 3.64] |
| YTHDF2 | GSE14814 | Lung cancer | NSCLC | DSS | JRB.10 | Zhu | 217812_at | 90 | 0.389 | 0.78 [0.45 - 1.37] |
| YTHDF2 | GSE14814 | Lung cancer | NSCLC | OS | JRB.10 | Zhu | 217812_at | 90 | 0.585 | 0.87 [0.52 - 1.44] |
| YTHDF2 | GSE4716-GPL3694 | Lung cancer | NSCLC | OS | Nagoya (1995-1996) | Tomida | 4921 | 50 | 0.757 | 1.23 [0.33 - 4.57] |
| YTHDF2 | GSE8894 | Lung cancer | NSCLC | RFS | Seoul (1995-2005) | Lee | 217812_at | 138 | 0.287 | 1.41 [0.75 - 2.65] |
| YTHDF2 | GSE8894 | Lung cancer | NSCLC | RFS | Seoul (1995-2005) | Lee | 222430_s_at | 138 | 0.175 | 1.42 [0.85 - 2.37] |
| YTHDF2 | GSE4573 | Lung cancer | Squamous cell carcinoma | OS | Michigan (1991-2002) | Raponi | 217812_at | 129 | 0.718 | 0.82 [0.29 - 2.36] |
| YTHDF2 | GSE17710 | Lung cancer | Squamous cell carcinoma | RFS | UNC | Wilkerson | 25693 | 56 | 0.482 | 0.73 [0.30 - 1.76] |
| YTHDF2 | GSE17710 | Lung cancer | Squamous cell carcinoma | OS | UNC | Wilkerson | 25693 | 56 | 0.687 | 0.83 [0.34 - 2.04] |
| YTHDF2 | GSE9891 | Ovarian cancer |  | OS | AOCS, RBH, WH, NKI-AVL (1992-2006) | Tothill | 217812_at | 278 | 0.117 | 1.43 [0.91 - 2.24] |
| YTHDF2 | GSE9891 | Ovarian cancer |  | OS | AOCS, RBH, WH, NKI-AVL (1992-2006) | Tothill | 222430_s_at | 278 | 0.337 | 1.22 [0.81 - 1.84] |
| YTHDF2 | DUKE-OC | Ovarian cancer |  | OS | Duke | Bild | 217812_at | 133 | 0.949 | 1.01 [0.68 - 1.51] |
| YTHDF2 | GSE26712 | Ovarian cancer |  | OS | MSKCC (1990-2003) | Bonome | 217812_at | 185 | 0.736 | 1.04 [0.83 - 1.30] |
| YTHDF2 | GSE26712 | Ovarian cancer |  | DFS | MSKCC (1990-2003) | Bonome | 217812_at | 185 | 0.673 | 1.04 [0.85 - 1.28] |
| YTHDF2 | GSE17260 | Ovarian cancer |  | PFS | Niigata (1997-2008) | Yoshihara | A_32_P99275 | 110 | 0.133 | 1.42 [0.90 - 2.26] |
| YTHDF2 | GSE17260 | Ovarian cancer |  | OS | Niigata (1997-2008) | Yoshihara | A_23_P103414 | 110 | 0.350 | 1.36 [0.71 - 2.58] |
| YTHDF2 | GSE17260 | Ovarian cancer |  | OS | Niigata (1997-2008) | Yoshihara | A_32_P99275 | 110 | 0.299 | 1.33 [0.78 - 2.29] |
| YTHDF2 | GSE17260 | Ovarian cancer |  | PFS | Niigata (1997-2008) | Yoshihara | A_23_P103414 | 110 | 0.363 | 1.30 [0.74 - 2.26] |
| YTHDF2 | GSE14764 | Ovarian cancer |  | OS | TOC | Denkert | 217812_at | 80 | 0.426 | 1.60 [0.50 - 5.13] |
| YTHDF2 | GSE19234 | Skin cancer | Melanoma | OS | NYU | Bogunovic | 217812_at | 38 | 0.229 | 2.58 [0.55 - 12.08] |
| YTHDF2 | GSE19234 | Skin cancer | Melanoma | OS | NYU | Bogunovic | 222430_s_at | 38 | 0.240 | 1.91 [0.65 - 5.64] |
| YTHDF2 | GSE30929 | Soft tissue cancer | Liposarcoma | DFS | MSKCC (1993-2008) | Gobble | 217812_at | 140 | 0.005 | 3.31 [1.45 - 7.56] |
| OS, Overall Survival;DSS,Disease Specific Survival;EFS,Event Free Survival;DFS,Disease Specific Survival;DMFS,Distant Metastasis Free Survival;RFS,Relapse Free Survival;PFS,Progression Free Survival;NSCLC, non-small cell lung carcinoma. | | | | | | | | | | |
|
|
|
|
|  |  |  |  |  |  |  |  |  |  |  |
