# Supplementary material for: YTHDF2 correlates with tumor immune infiltrates in lower-grade glioma
Source: Aging (Albany NY). 2020 Sep 27;12(18):18476–500. doi: 10.18632/aging.103812 (PMC7585119; doi:10.18632/aging.103812)
Supplement: Supplementary Table 3 [file aging-12-103812-s003..doc]

| Supplementary Table 3. Prognostic values of YTHDF2 in cancers analyzed by GEPIA, TIMER, OncoLnc and Kaplan-Meier plotter. | | | | | | | | | | | | | |
| --- | --- | --- | --- | --- | --- | --- | --- | --- | --- | --- | --- | --- | --- |
|
| GEPIA | | | | | TIMER | | | OncoLnc | | | Kaplan-Meier plotter | | |
|  | OS | | DFS | |  | OS | |  | OS | |  | OS | |
| Cancer | HR | P-value | HR | P-value | Cancer | HR(95%CI) | P-value | Cancer | Cox Coefficient | P-value | Cancer | HR(95%CI) | P-value |
| ACC | 2.1 | 0.065 | 1.9 | 0.054 | ACC | 2.239 (0.647-7.744) | 0.203 | BLCA | -0.064 | 0.38 | BLCA | 0.69 (0.51-0.92) | * |
| BLCA | 0.91 | 0.53 | 1.2 | 0.32 | BLCA | 0.757 (0.508-1.129) | 0.172 | BRCA | 0.04 | 0.68 | BRCA | 0.74 (0.51-1.08) | 0.11 |
| BRCA | 1.1 | 0.5 | 0.88 | 0.5 | BRCA | 0.915 (0.578-1.448) | 0.704 | CESC | 0.009 | 0.94 | CESC | 1.76 (0.98-3.17) | 0.055 |
| CESC | 1.4 | 0.15 | 1 | 0.94 | CESC | 1.209 (0.636-2.298) | 0.562 | COAD | -0.139 | 0.16 | ESCA | 0.69 (0.41-1.17) | 0.17 |
| CHOL | 0.77 | 0.6 | 0.53 | 0.18 | CHOL | 1.039 (0.254-1.259) | 0.958 | ESCA | -0.036 | 0.8 | HNSC | 0.8 (0.59-1.08) | 0.15 |
| COAD | 0.75 | 0.24 | 0.93 | 0.77 | COAD | 0.721 (0.423-1.228) | 0.958 | GBM | 0.031 | 0.73 | KIRC | 0.58 (0.43-0.78) | *** |
| DLBC | 2.2 | 0.29 | 2.1 | 0.22 | DLBC | 4.037 (0.254-64.2) | 0.323 | HNSC | 0.001 | 0.99 | KIRP | 0.69 (0.35-1.37) | 0.29 |
| ESCA | 0.63 | 0.053 | 1.3 | 0.24 | ESCA | 0.86 (0.478-1.547) | 0.615 | KIRC | -0.051 | 0.53 | LIHC | 2.71 (1.9-3.87) | *** |
| GBM | 1.1 | 0.46 | 0.83 | 0.39 | GBM | 0.992 (0.834-1.18) | 0.932 | KIRP | -0.044 | 0.78 | LUAD | 0.67 (0.5-0.9) | ** |
| HNSC | 0.99 | 0.95 | 0.85 | 0.35 | HNSC | 0.961 (0.631-1.462) | 0.852 | LAML | -0.027 | 0.8 | LUSC | 1.16 (0.85-1.58) | 0.36 |
| KICH | 9.2 | * | 4.7 | * | KICH | 24.208 (2.122-276.177) | * | LGG | 0.329 | *** | OV | 0.73 (0.56-0.95) | * |
| KIRC | 0.63 | ** | 0.63 | * | KIRC | 0.826 (0.483-1.413) | 0.485 | LIHC | 0.316 | *** | PAAD | 1.4 (0.87-2.26) | 0.17 |
| KIRP | 1 | 0.98 | 1.1 | 0.69 | KIRP | 0.941 (0.369-2.396) | 0.898 | LUAD | -0.128 | 0.095 | PCPG | 3.08 (0.54-17.55) | 0.18 |
| LAML | 1.1 | 0.73 | 1 | 1 | LAML | No data | no data | LUSC | -0.005 | 0.94 | READ | 0.47 (0.22-1.01) | * |
| LGG | 1.8 | ** | 2 | *** | LGG | 2.749 (1.697-4.453) | *** | OV | 0.027 | 0.73 | SARC | 2.71 (1.62-4.55) | *** |
| LIHC | 1.6 | ** | 1.3 | 0.081 | LIHC | 2.194 (1.334-3.608) | ** | PAAD | 0.065 | 0.52 | STAD | 0.78 (0.56-1.08) | 0.13 |
| LUAD | 0.87 | 0.36 | 0.89 | 0.45 | LUAD | 0.742 (0.493-1.116) | 0.152 | READ | -0.53 | * | TGCT | 0 (0-inf) | 0.064 |
| LUSC | 1 | 0.87 | 0.98 | 0.88 | LUSC | 0.997 (0.694-1.431) | 0.985 | SARC | 0.428 | *** | THYM | 0 (0-inf) | * |
| MESO | 1 | 1 | 1 | 0.87 | MESO | 0.605 (0.308-1.185) | 0.143 | SKCM | 0.005 | 0.94 | THCA | 2.28 (0.85-6.15) | 0.093 |
| OV | 1.2 | 0.15 | 1 | 0.7 | OV | 1.013 (0.68-1.508) | 0.95 | STAD | -0.081 | 0.34 | UCEC | 0.73 (0.47-1.12) | 0.15 |
| PAAD | 1 | 0.93 | 1.4 | 0.14 | PAAD | 1.37 (0.68-2.75) | 0.373 | UCEC | -0.173 | 0.08 |  |  |  |
| PCPG | 1.1 | 0.92 | 1.3 | 0.63 | PCPG | 1.032 (0.124-8.577) | 0.977 |  |  |  |  |  |  |
| PRAD | 1.7 | 0.42 | 1.2 | 0.5 | PRAD | 11.714 (0.611-224.408) | 0.102 |  |  |  |  |  |  |
| READ | 0.38 | 0.054 | 1.1 | 0.88 | READ | 0.418 (0.15-1.16) | 0.094 |  |  |  |  |  |  |
| SARC | 2.1 | *** | 1.3 | 0.16 | SARC | 3.024 (1.725-5.302) | *** |  |  |  |  |  |  |
| SKCM | 1.1 | 0.36 | 1 | 0.88 | SKCM | 0.93 (0.721-1.199) | 0.574 |  |  |  |  |  |  |
| STAD | 1.1 | 0.6 | 0.75 | 0.14 | STAD | 0.724 (0.433-1.211) | 0.218 |  |  |  |  |  |  |
| TGCT | 0.95 | 0.96 | 0.81 | 0.54 | TGCT | 0.418 (0.051-3.408) | 0.415 |  |  |  |  |  |  |
| THCA | 1.3 | 0.59 | 0.87 | 0.62 | THCA | 1.876 (0.103-34.309) | 0.671 |  |  |  |  |  |  |
| THYM | 0.81 | 0.77 | 1.2 | 0.73 | THYM | 0.449 (0.03-6.623) | 0.56 |  |  |  |  |  |  |
| UCEC | 0.48 | * | 0.63 | 1.6 | UCEC | 0.73 (0.408-1.306) | 0.29 |  |  |  |  |  |  |
| UCS | 1.2 | 0.64 | 1.1 | 0.86 | UCS | 1.253 (0.573-2.743) | 0.572 |  |  |  |  |  |  |
| UVM | 1.5 | 0.42 | 1.3 | 0.55 | UVM | 1.393 (0.648-2.996) | 0.396 |  |  |  |  |  |  |
| ACC,Adrenocortical carcinoma; BLCA,Bladder Urothelial Carcinoma; BRCA,Breast invasive carcinoma; CESC,Cervical squamous cell carcinoma and endocervical adenocarcinoma; CHOL,Cholangio carcinoma; COAD,Colon adenocarcinoma; DLBC Lymphoid Neoplasm Diffuse Large B-cell Lymphoma; ESCA,Esophageal carcinoma; GBM,Glioblastoma multiforme; HNSC,Head and Neck squamous cell carcinoma; KICH,Kidney Chromophobe; KIRC,Kidney renal clear cell carcinoma; KIRP, Kidney renal papillary cell carcinoma; LAML,Acute Myeloid Leukemia; LGG,Brain Lower Grade Glioma; LIHC,Liver hepatocellular carcinoma; LUAD,Lung adenocarcinoma; LUSC,Lung squamous cell carcinoma; MESO,Mesothelioma; OV,Ovarian serous cystadenocarcinoma; PAAD,Pancreatic adenocarcinoma; PCPG,Pheochromocytoma and Paraganglioma; PRAD,Prostate adenocarcinoma; READ,Rectum adenocarcinoma; SARC,Sarcoma; SKCM,Skin Cutaneous Melanoma; STAD,Stomach adenocarcinoma; TGCT,Testicular Germ Cell Tumors; THCA,Thyroid carcinoma; THYM,Thymoma; UCEC,Uterine Corpus Endometrial Carcinoma; UCS,Uterine Carcinosarcoma; UVM,Uveal Melanoma. P-value Significant Codes: 0 ≤ *** < 0.001 ≤ ** < 0.01 ≤ * < 0.05. | | | | | | | | | | | | | |
|
|
|
|
|
|
|  |  |  |  |  |  |  |  |  |  |  |  |  |  |
