# Supplementary material for: YTHDF2 correlates with tumor immune infiltrates in lower-grade glioma
Source: Aging (Albany NY). 2020 Sep 27;12(18):18476–500. doi: 10.18632/aging.103812 (PMC7585119; doi:10.18632/aging.103812)
Supplement: Supplementary Table 4 [file aging-12-103812-s006..pdf]

SUPPLEMENTARY TABLE

Supplementary Table 4. The expression level and survival analysis of YTHDF2 with different clinicopathological characteristics in LGG,LIHC and SARC (UALCAN database).

	LGG			LIHC		SARC	
**Expression analysis**		**P-value**			**P-value**		**P-value**
**Sample Type**							
Normal-vs-Primary tumor	NA			High	3.63E-12	Not sig	2.39E-01
**Gender**							
Normal-vs-Male	NA			high	8.52E-11	Not sig	3.05E-01
Normal-vs-Female	NA			high	4.73E-10	Not sig	1.90E-01
Male-vs-Female	Not sig	3.06E-01		Not sig	5.46E-01	high	4.22E-03
**Tumor grade**							
Normal-vs-Grade 1	NA			high	5.93E-05	NA	
Normal-vs-Grade 2	NA			high	4.07E-08	NA	
Normal-vs-Grade 3	NA			high	1.23E-11	NA	
Normal-vs-Grade 4	NA			high	1.89E-02	NA	
Grade 1-vs-Grade 2	NA			Not sig	8.68E-01	NA	
Grade 1-vs-Grade 3	NA			high	1.65E-02	NA	
Grade 1-vs-Grade 4	NA			Not sig	2.19E-01	NA	
Grade 2-vs-Grade 3	high	1.10E-06		high	7.18E-03	NA	
Grade 2-vs-Grade 4	NA			Not sig	8.90E-02	NA	
Grade 3-vs-Grade 4	NA				6.75E-01	NA	
**TP53 mutation status**							
Normal-vs-TP53-Mutant	NA			high	1.62E-12	Not sig	3.05E-01
Normal-vs-TP53-NonMutant	NA			high	1.63E-07	Not sig	2.09E-01
TP53-Mutant-vs-TP53-NonMutant	low	1.62E-12		low	1.06E-08	Not sig	8.60E-01
**Histological subtypes**							
Astrocytoma-vs-Oligoastrocytoma	low	7.22E-04	Normal-vs-N0	high	3.65E-12	NA	
Astrocytoma-vs-Oligodendroglioma	low	3.44E-12	Normal-vs-N1	high	3.08E-03	NA	
Oligoastrocytoma-vs-Oligodendroglioma	low	1.32E-03	N0-vs-N1	Not sig	8.31E-01	NA	
**Survival analysis**							
Expression level	sig	P<0.0001		sig	P<0.0001	sig	P=0.0094
Tumor grade	sig	P<0.0001		sig	P<0.0001	NA	
Gender	sig	P=0.0075		sig	P<0.0001	sig	P=0.032

Note: LGG, Brain Lower Grade Glioma; LIHC, Liver hepatocellular carcinoma; SARC, Sarcoma; YTHDF2, YTH N6-methyladenosine RNA binding protein 2; high, means high expression; low, means low expression; sig, means significant; Not sig, means not significant; NA, means not available; Grade 1, Well differentiated (low grade); Grade 2, Moderately differentiated (intermediate grade); Grade 3, Poorly differentiated (high grade); Grade 4, Undifferentiated (high grade); N0,

No regional lymph node metastasis; N1, Metastases in 1 to 3 axillary lymph nodes; N2, Metastases in 4 to 9 axillary lymph nodes; N3, Metastases in 10 or more axillary lymph nodes.
