# Supplementary material for: YTHDF2 correlates with tumor immune infiltrates in lower-grade glioma
Source: Aging (Albany NY). 2020 Sep 27;12(18):18476–500. doi: 10.18632/aging.103812 (PMC7585119; doi:10.18632/aging.103812)
Supplement: Supplementary Table 5 [file aging-12-103812-s002..doc]

| Supplementary Table 5. Correlation analysis between YTHDF2 and immune infiltration level in cancers by TIMER. | | | | | | | | | | | | | | |
| --- | --- | --- | --- | --- | --- | --- | --- | --- | --- | --- | --- | --- | --- | --- |
|
|  | Purity | | B Cell | | CD8+ T Cell | | CD4+ T Cell | | Macrophage | | Neutrophil | | Dendritic Cell | |
| **Cancer** | cor | p | cor | p | cor | p | cor | p | cor | p | cor | p | cor | p |
| ACC | -0.014 | 0.9027 | -0.067 | 0.5722 | -0.001 | 0.9955 | 0.122 | 0.3035 | -0.146 | 0.218 | -0.188 | 0.111 | -0.052 | 0.6593 |
| BLCA | 0.323 | *** | 0.028 | 0.5889 | -0.077 | 0.142 | -0.047 | 0.3674 | -0.173 | *** | -0.071 | 0.1761 | -0.124 | 0.0179 |
| BRCA | 0.062 | 0.05 | 0.007 | 0.8155 | 0.047 | 0.1461 | -0.097 | ** | -0.107 | *** | -0.052 | 0.1091 | -0.091 | ** |
| CESC | 0.050 | 0.4106 | -0.111 | 0.0658 | 0.085 | 0.1586 | -0.002 | 0.9777 | 0.050 | 0.4063 | 0.030 | 0.6222 | 0.089 | 0.1409 |
| CHOL | -0.330 | * | 0.057 | 0.7458 | -0.095 | 0.5855 | -0.184 | 0.2911 | -0.013 | 0.9411 | -0.039 | 0.8249 | 0.000 | 0.9987 |
| COAD | 0.015 | 0.7584 | 0.167 | *** | 0.263 | *** | -0.184 | *** | -0.092 | 0.0661 | 0.059 | 0.2409 | 0.040 | 0.4217 |
| DLBC | 0.057 | 0.7219 | 0.391 | 0.1084 | 0.110 | 0.6348 | 0.051 | 0.8247 | -0.048 | 0.8352 | 0.458 | * | 0.016 | 0.9463 |
| ESCA | 0.112 | 0.1325 | 0.018 | 0.8153 | -0.013 | 0.8664 | -0.040 | 0.592 | -0.229 | ** | 0.112 | 0.1339 | -0.059 | 0.4277 |
| GBM | 0.206 | *** | 0.027 | 0.5766 | 0.063 | 0.2 | -0.005 | 0.9121 | 0.189 | *** | 0.225 | *** | 0.113 | * |
| HNSC | -0.073 | 0.1047 | -0.110 | * | -0.093 | * | -0.121 | ** | -0.220 | *** | -0.026 | 0.564 | -0.141 | ** |
| KICH | -0.173 | 0.1657 | 0.271 | * | 0.004 | 0.9746 | 0.141 | 0.2639 | 0.129 | 0.3066 | 0.291 | * | 0.350 | ** |
| KIRC | -0.185 | *** | 0.043 | 0.3544 | 0.088 | 0.0663 | -0.011 | 0.819 | 0.047 | 0.318 | 0.043 | 0.3597 | 0.055 | 0.2379 |
| KIRP | -0.010 | 0.8717 | 0.119 | 0.0563 | 0.068 | 0.2766 | -0.034 | 0.5907 | -0.054 | 0.3942 | 0.152 | * | 0.085 | 0.1775 |
| LGG | -0.142 | ** | 0.505 | *** | 0.250 | *** | 0.379 | *** | 0.309 | *** | 0.468 | *** | 0.489 | *** |
| LIHC | -0.143 | ** | -0.046 | 0.3998 | 0.021 | 0.6978 | 0.021 | 0.7 | 0.048 | 0.376 | 0.159 | ** | 0.053 | 0.3297 |
| LUAD | 0.130 | ** | -0.075 | 0.1007 | -0.132 | ** | -0.186 | *** | -0.194 | *** | -0.187 | *** | -0.166 | *** |
| LUSC | -0.057 | 0.2113 | 0.070 | 0.13 | -0.048 | 0.3014 | -0.008 | 0.8678 | -0.045 | 0.3223 | -0.007 | 0.8811 | -0.068 | 0.142 |
| MESO | -0.138 | 0.2058 | 0.004 | 0.9697 | 0.066 | 0.5513 | -0.253 | * | -0.183 | 0.0962 | 0.056 | 0.6133 | 0.059 | 0.5951 |
| OV | 0.154 | *** | 0.068 | 0.1358 | 0.064 | 0.162 | 0.002 | 0.9655 | 0.068 | 0.1347 | 0.059 | 0.1976 | 0.070 | 0.1254 |
| PAAD | -0.094 | 0.2218 | 0.146 | 0.0573 | 0.187 | * | -0.047 | 0.54 | 0.061 | 0.4306 | 0.121 | 0.1155 | 0.187 | * |
| PCPG | -0.024 | 0.7559 | 0.182 | * | 0.448 | *** | 0.038 | 0.6242 | 0.168 | * | 0.158 | * | 0.114 | 0.1428 |
| PRAD | 0.176 | *** | -0.204 | *** | -0.141 | ** | -0.274 | *** | -0.407 | *** | -0.144 | ** | -0.171 | *** |
| READ | 0.035 | 0.6782 | -0.103 | 0.2289 | 0.260 | ** | -0.133 | 0.1197 | -0.112 | 0.1895 | 0.042 | 0.6277 | -0.145 | 0.0894 |
| SARC | -0.181 | ** | -0.030 | 0.6477 | -0.044 | 0.4973 | -0.115 | 0.0764 | -0.030 | 0.6461 | -0.066 | 0.3077 | -0.031 | 0.6275 |
| SKCM | 0.218 | *** | 0.039 | 0.4124 | 0.241 | *** | -0.067 | 0.1572 | -0.020 | 0.6664 | 0.169 | *** | 0.015 | 0.7559 |
| STAD | 0.149 | ** | -0.053 | 0.3059 | -0.094 | 0.0716 | -0.179 | *** | -0.258 | *** | -0.097 | 0.0624 | -0.160 | ** |
| TGCT | -0.185 | * | 0.134 | 0.1055 | 0.316 | *** | -0.417 | *** | -0.242 | ** | -0.260 | ** | -0.039 | 0.6432 |
| THCA | -0.066 | 0.1438 | 0.122 | ** | 0.050 | 0.274 | 0.174 | *** | 0.189 | *** | 0.198 | *** | 0.184 | *** |
| THYM | -0.090 | 0.3393 | 0.401 | *** | 0.480 | *** | 0.396 | *** | 0.231 | * | -0.098 | 0.2981 | 0.483 | *** |
| UCEC | 0.126 | * | -0.176 | ** | 0.088 | 0.1351 | -0.229 | *** | -0.048 | 0.4179 | 0.015 | 0.8015 | -0.152 | ** |
| UCS | -0.051 | 0.7167 | -0.131 | 0.3509 | 0.118 | 0.4009 | -0.273 | * | -0.221 | 0.1118 | -0.045 | 0.7492 | -0.102 | 0.4684 |
| UVM | 0.057 | 0.6232 | 0.397 | *** | 0.371 | *** | -0.287 | * | -0.265 | * | -0.072 | 0.5335 | 0.015 | 0.8999 |
| ACC,Adrenocortical carcinoma; BLCA,Bladder Urothelial Carcinoma; BRCA,Breast invasive carcinoma; CESC,Cervical squamous cell carcinoma and endocervical adenocarcinoma; CHOL,Cholangio carcinoma; COAD,Colon adenocarcinoma; DLBC Lymphoid Neoplasm Diffuse Large B-cell Lymphoma; ESCA,Esophageal carcinoma; GBM,Glioblastoma multiforme; HNSC,Head and Neck squamous cell carcinoma; KICH,Kidney Chromophobe; KIRC,Kidney renal clear cell carcinoma; KIRP, Kidney renal papillary cell carcinoma; LAML,Acute Myeloid Leukemia; LGG,Brain Lower Grade Glioma; LIHC,Liver hepatocellular carcinoma; LUAD,Lung adenocarcinoma; LUSC,Lung squamous cell carcinoma; MESO,Mesothelioma; OV,Ovarian serous cystadenocarcinoma; PAAD,Pancreatic adenocarcinoma; PCPG,Pheochromocytoma and Paraganglioma; PRAD,Prostate adenocarcinoma; READ,Rectum adenocarcinoma; SARC,Sarcoma; SKCM,Skin Cutaneous Melanoma; STAD,Stomach adenocarcinoma; TGCT,Testicular Germ Cell Tumors; THCA,Thyroid carcinoma; THYM,Thymoma; UCEC,Uterine Corpus Endometrial Carcinoma; UCS,Uterine Carcinosarcoma; UVM,Uveal Melanoma. P-value Significant Codes: 0 ≤ *** < 0.001 ≤ ** < 0.01 ≤ * < 0.05. | | | | | | | | | | | | | | |
|
|
|
|
|
|  |  |  |  |  |  |  |  |  |  |  |  |  |  |  |
