# Supplementary material for: YTHDF2 correlates with tumor immune infiltrates in lower-grade glioma
Source: Aging (Albany NY). 2020 Sep 27;12(18):18476–500. doi: 10.18632/aging.103812 (PMC7585119; doi:10.18632/aging.103812)
Supplement: Supplementary Tables 6, 7 and 8 [file aging-12-103812-s005..pdf]

SUPPLEMENTARY TABLES

Supplementary Table 6. Prognostic values of relate genes and markers of TAMs in LGG analyzed by GEPIA.

Description	Gene markers	LGG			
		OS		DFS	
		HR	P-value	HR	P-value
TAM	CCL2	1.40	0.05100	1.00	0.96000
	CSF1	1.00	0.86000	1.20	0.32000
	CSF1R	1.20	0.37000	1.20	0.35000
	EGF	1.90	***	1.50	**
	STAT3	1.90	***	1.60	**
	STAT6	1.90	***	1.40	*
	IL6	1.90	***	1.40	*
	IL10	1.60	**	1.30	0.16000
	TLR4	1.00	0.94000	0.96	0.82000
	TGFβ (TGFB1)	1.70	**	1.50	**
	LOX	3.20	***	1.70	**
	PD-L1(CD274)	1.80	**	1.30	0.12000
	PD-L2(PDCD1LG2)	2.00	***	2.00	***
	CD80	2.50	***	1.60	**
	CD86	1.80	**	1.40	*
	MFGE8	1.10	0.68000	0.81	0.17000

LGG, Brain Lower Grade Glioma.P-value Significant Codes: 0 ≤ *** < 0.001 ≤ ** < 0.01 ≤ * < 0.05.

Supplementary Table 7. Correlation analysis between YTHDF2 and relate genes and markers of TAMs in LAML by GEPIA.

Description	Gene markers	LAML	
		Cor	p-value
TAMs	CCL2	-0.003	0.9700
	CSF1	0.240	**
	CSF1R	-0.018	0.8100
	EGF	0.210	**
	STAT3	0.430	***
	STAT6	0.230	**
	IL6	0.002	0.9800
	IL10	-0.079	0.3000
	TLR4	-0.028	0.7100
	TGFβ (TGFB1)	0.160	*
	LOX	0.270	***
	PD-L1(CD274)	0.200	**
	PD-L2(PDCD1LG2)	-0.035	0.6500
	CD80	0.280	***
	CD86	-0.240	**
	MFGE8	0.480	***

LAML, Acute Myeloid Leukemia; TAMs, tumor-associated macrophages; Cor, R value of Spearman's correlation; P-value Significant Codes: 0 ≤ *** < 0.001 ≤ ** < 0.01 ≤ * < 0.05.

Supplementary Table 8. The enrichment analysis of YTHDF2 in LGG by GSEA tool of LinkedOmics database.

	Gene Set	Description	Size	P-Value
**BP**	GO:0006302	double-strand break repair	56	0
	GO:0071166	ribonucleoprotein complex localization	41	0
	GO:0006260	DNA replication	90	0
	GO:0000075	cell cycle checkpoint	75	0
	GO:0044772	mitotic cell cycle phase transition	156	0
	GO:0006333	chromatin assembly or disassembly	55	0
	GO:0016458	gene silencing	55	0
	GO:0006338	chromatin remodeling	55	0
	GO:0040029	regulation of gene expression, epigenetic	75	0
	GO:0006403	RNA localization	75	0
**CC**	GO:0044815	DNA packaging complex	21	0
	GO:0032993	protein-DNA complex	58	0
	GO:0016607	nuclear speck	108	0
	GO:0005657	replication fork	25	0
	GO:0098687	chromosomal region	100	0
	GO:0035145	exon-exon junction complex	5	0.005
	GO:0017053	transcriptional repressor complex	25	0.012
	GO:0000793	condensed chromosome	61	0.008
	GO:0016605	PML body	32	0.011
	GO:0034399	nuclear periphery	51	0.004
**MF**	GO:0001085	RNA polymerase II transcription factor binding	51	0
	GO:0070491	repressing transcription factor binding	25	0
	GO:0051059	NF-kappaB binding	8	0
	GO:0031491	nucleosome binding	27	0
	GO:0043178	alcohol binding	24	0
	GO:0017056	structural constituent of nuclear pore	12	0.004
	GO:0035326	enhancer binding	43	0
	GO:0042826	histone deacetylase binding	35	0.007
	GO:0008327	methyl-CpG binding	9	0
	GO:0003714	transcription corepressor activity	74	0.004
**KEGG Pathway**	hsa04110	Cell cycle	46	0
	hsa03040	Spliceosome	40	0
	hsa03013	RNA transport	56	0
	hsa04152	AMPK signaling pathway	38	0
	hsa00020	Citrate cycle (TCA cycle)	9	0
	hsa05203	Viral carcinogenesis	63	0.004
	hsa03430	Mismatch repair	8	0.012
	hsa05166	Human T-cell leukemia virus 1 infection	81	0.025
	hsa03030	DNA replication	13	0.038
**Panther Pathway**	P00020	FAS signaling pathway	17	0.004
	P00014	Cholesterol biosynthesis	6	0.004
	P00017	DNA replication	8	0.021
	P02762	Pentose phosphate pathway	4	0.023
	P02746	Heme biosynthesis	5	0.031
	P00016	Cytoskeletal regulation by Rho GTPase	23	0.032
	P00053	T cell activation	34	0.035
**Reacmoe Pathway**	R-HSA-8936459	RUNX1 regulates genes involved in megakaryocyte differentiation and platelet function	33	0
	R-HSA-5250913	Positive epigenetic regulation of rRNA expression	37	0
	R-HSA-73728	RNA Polymerase I Promoter Opening	24	0
	R-HSA-201722	Formation of the beta-catenin:TCF transactivating complex	33	0
	R-HSA-3247509	Chromatin modifying enzymes	74	0
	R-HSA-427359	SIRT1 negatively regulates rRNA expression	26	0
	R-HSA-1912408	Pre-NOTCH Transcription and Translation	34	0
	R-HSA-1640170	Cell Cycle	196	0
	R-HSA-71403	Citric acid cycle (TCA cycle)	4	0.004
	R-HSA-9604323	Negative regulation of NOTCH4 signaling	15	0.016

**Wiki Pathway**	WP314	Fas Ligand (FasL) pathway and Stress induction of Heat Shock Proteins (HSP) regulation	25	0
	WP411	mRNA Processing	35	0
	WP2446	Retinoblastoma Gene in Cancer	32	0
	WP466	DNA Replication	13	0
	WP179	Cell Cycle	44	0
	WP78	TCA Cycle (aka Krebs or citric acid cycle)	4	0
	WP531	DNA Mismatch Repair	8	0.008
	WP2453	TCA Cycle and Deficiency of Pyruvate Dehydrogenase complex (PDHc)	6	0.036
	WP1742	TP53 Network	12	0.038
	WP3664	Regulation of Wnt/B-catenin Signaling by Small Molecule Compounds	4	0.048

Note: LGG, Brain Lower Grade Glioma; YTHDF2, YTH N6-methyladenosine RNA binding protein 2; GSEA, Gene Set Enrichment Analysis; BP, biological process; CC, cellular component; MF, molecular function; GO, Gene Ontology; KEGG, Kyoto Encyclopedia of Genes and Genomes.
